# Kelp carbon sink potential decreases with warming due to accelerating decomposition

**DOI:** 10.1371/journal.pbio.3001702

**Published:** 2022-08-04

**Authors:** Karen Filbee-Dexter, Colette J. Feehan, Dan A. Smale, Kira A. Krumhansl, Skye Augustine, Florian de Bettignies, Michael T. Burrows, Jarrett E. K. Byrnes, Jillian Campbell, Dominique Davoult, Kenneth H. Dunton, João N. Franco, Ignacio Garrido, Sean P. Grace, Kasper Hancke, Ladd E. Johnson, Brenda Konar, Pippa J. Moore, Kjell Magnus Norderhaug, Alasdair O’Dell, Morten F. Pedersen, Anne K. Salomon, Isabel Sousa-Pinto, Scott Tiegs, Dara Yiu, Thomas Wernberg

**Affiliations:** 1 Institute of Marine Research, His, Norway; 2 UWA Oceans Institute & School of Biological Sciences, The University of Western Australia, Perth, Australia; 3 Department of Biology, Montclair State University, Montclair, New Jersey, United States of America; 4 Marine Biological Association of the United Kingdom, The Laboratory, Plymouth, United Kingdom; 5 Bedford Institute of Oceanography, Fisheries and Oceans Canada, Dartmouth, Nova Scotia, Canada; 6 Department of Biology, University of Victoria, Victoria, British Columbia, Canada; 7 Sorbonne Université, CNRS, Station Biologique de Roscoff, Place Georges Teissier, Roscoff, France; 8 Scottish Association for Marine Science, Oban, Argyll, Scotland; 9 Department of Biology, University of Massachusetts Boston, Boston, Massachusetts, United States of America; 10 Marine Science Institute, The University of Texas at Austin, Port Aransas, Texas, United States of America; 11 Marine and Environmental Sciences Centre, ESTM, Politécnico de Leiria, Peniche, Portugal; 12 CIIMAR—Interdisciplinary Centre of Marine and Environmental Research, and Faculty of Sciences, University of Porto, Porto, Portugal; 13 Department of Biology and Québec-Océan, Laval University, Québec, Québec, Canada; 14 Centro FONDAP de Investigación en Dinámica de Ecosistemas Marinos de Altas Latitudes (IDEAL), Facultad de Ciencias, Universidad Austral de Chile (UACh), Valdivia, Chile; 15 Department of Biology, Werth Center for Coastal and Marine Studies, Southern Connecticut State University, New Haven, Connecticut, United States of America; 16 Norwegian Institute for Water Research (NIVA), Section for Marine Biology, Oslo, Norway; 17 College of Fisheries and Ocean Sciences, University of Alaska Fairbanks, Fairbanks, Alaska, United States of America; 18 The Dove Marine Laboratory, School of Natural and Environmental Science, Newcastle University, Newcastle, United Kingdom; 19 Department of Science and Environment, Roskilde University, Roskilde, Denmark; 20 Oakland University, Department of Biological Sciences, Michigan, United States of America; 21 University of Washington, School of Aquatic and Fishery Sciences, Seattle, Washington, United States of America; University of Cambridge, UNITED KINGDOM

## Abstract

Cycling of organic carbon in the ocean has the potential to mitigate or exacerbate global climate change, but major questions remain about the environmental controls on organic carbon flux in the coastal zone. Here, we used a field experiment distributed across 28° of latitude, and the entire range of 2 dominant kelp species in the northern hemisphere, to measure decomposition rates of kelp detritus on the seafloor in relation to local environmental factors. Detritus decomposition in both species were strongly related to ocean temperature and initial carbon content, with higher rates of biomass loss at lower latitudes with warmer temperatures. Our experiment showed slow overall decomposition and turnover of kelp detritus and modeling of coastal residence times at our study sites revealed that a significant portion of this production can remain intact long enough to reach deep marine sinks. The results suggest that decomposition of these kelp species could accelerate with ocean warming and that low-latitude kelp forests could experience the greatest increase in remineralization with a 9% to 42% reduced potential for transport to long-term ocean sinks under short-term (RCP4.5) and long-term (RCP8.5) warming scenarios. However, slow decomposition at high latitudes, where kelp abundance is predicted to expand, indicates potential for increasing kelp-carbon sinks in cooler (northern) regions. Our findings reveal an important latitudinal gradient in coastal ecosystem function that provides an improved capacity to predict the implications of ocean warming on carbon cycling. Broad-scale patterns in organic carbon decomposition revealed here can be used to identify hotspots of carbon sequestration potential and resolve relationships between carbon cycling processes and ocean climate at a global scale.

## Abstract 2

Cycling of organic carbon in the ocean has the potential to mitigate or exacerbate global climate change, but how is this influenced by the environment? A field experiment at 35 sites spanning 12 geographic regions allows measurement of decomposition rates and changes in carbon content of kelp detritus in coastal habitats, revealing a strong influence of an ocean-temperature gradient on decomposition.

## Introduction

The cycling of organic carbon in the coastal ocean is a critical yet unresolved component of the global carbon cycle [1,2]. Consequently, there has been a strong focus on resolving inorganic carbon (CO_2_) uptake and primary productivity on global scales [3]. Yet, decomposition rates of organic carbon at the ecosystem scale, which are known to vary with environmental conditions such as temperature (e.g., [4,5]), could be equally important in determining the balance between pools of organic and inorganic carbon [6–8]. At the land–sea interface, carbon cycling by macroalgae and other macrophytes has recently emerged as an important process by which CO_2_ is captured, stored, and potentially sequestered in the ocean through transport to deep marine sediments [9,10]. Macroalgal forests are the largest marine biome in the world, covering 1.5 to 2 million km^2^ [11] with a high productivity (average 516 g C m^−2^ y^−1^; [12]). Coarse estimates suggest they could sequester 173 Tg C yr^−1^ [9], which is almost double that sequestered by mangrove, saltmarsh, and seagrass “blue carbon habitats” combined (IPCC 2022). As such, quantifying rates of decomposition of macroalgal detritus in the marine environment is essential to estimate its potential contribution to blue carbon (carbon captured by ocean and coastal ecosystems) [13] and its uptake by coastal food webs and fate in the global carbon cycle more generally.

Decomposition rates of organic carbon vary geographically, and this is a challenge for current climate models, which usually use spatially uniform relationships to represent major processes or pathways [1,14–16]. On land, models that consider spatiotemporal dependencies in temperature, microbial, and mineral surface interactions predict weaker and more variable soil-carbon–climate feedbacks than models using average rates [17]. In the open ocean, the global biological pump has large regional variability, with particulate organic carbon (POC) decomposition rates ranging over 2 orders of magnitude [18,19]. As a result of these spatial differences, commonly applied rates of POC decomposition based on measures from a few areas have overestimated the global flux of POC to the seafloor [18]. Similarly, variation in deep sea benthic communities appears to drive strong heterogeneity in carbon turnover rates following deposition [1] and latitudinal differences in microbial activity are expected to drive slower degradation rates of dissolved organic carbon at higher latitudes [4].

The dynamics of temperature–decomposition relationships are also complex [20]. Organic matter tends to be remineralized faster in warmer low latitude environments compared to cooler high latitude environments (i.e., Arrhenius theory) [21,22], and the temperature-dependent decomposition of carbon has been highlighted as a key source of uncertainty in future global carbon models [5,23,24]. Understanding the environmental drivers underlying spatial variation in carbon turnover is critical because it effectively controls how current rates of carbon cycling might change with global warming. In particular, it informs whether environmental and biological changes will create positive feedbacks on the entire carbon cycle that lead to further warming, as opposed to negative feedbacks that buffer impacts and buy time to reduce emissions.

Large brown macroalgae form kelp forests along temperate coasts, assimilating substantial quantities of CO_2_ by virtue of their exceptional productivity and large spatial extent [25,26]. Many kelp forests have declined or are predicted to decline globally, particularly in regions with high seawater temperatures and rapid warming [26–30]. In contrast, it appears many kelp forests in cooler regions are relatively stable, and in some cases, kelp could even be increasing in abundance [29,31–33]. Changes in the abundance of kelp, and the environmental conditions they experience, may have consequences for the global carbon cycle. More than 80% of kelp production enters the coastal ecosystem as detritus, where it eventually strands on beaches, sinks to the seafloor, or is consumed or decomposed [25,34]. In general, the slower the decomposition of kelp detritus in the ocean, the greater chance it has for long-term storage in the deep ocean and the longer it takes to reenter the atmosphere as CO_2_ [18,35]. For example, macroalgal detritus that reaches the deeper ocean below the mixed layer is considered trapped in water masses where the CO_2_ is retained for significant time periods (i.e., >1,000 years) before returning to the ocean surface and eventually the atmosphere [9,36]. Detritus that is retained in some nearshore areas, such as deep fjords or basins with high rates of sedimentation, may also be buried for 100s to 1,000s of years, effectively removing it from the short-term carbon cycle [37–40]. Conversely, detritus that is decomposed rapidly has little chance of reaching these deep marine sinks, but instead can rapidly enter coastal food webs as a resource subsidy. In this context, knowledge of the rates and drivers of kelp decomposition in the coastal zone is required to better understand the role of kelp forests in the global carbon cycle.

**Fig 1:**
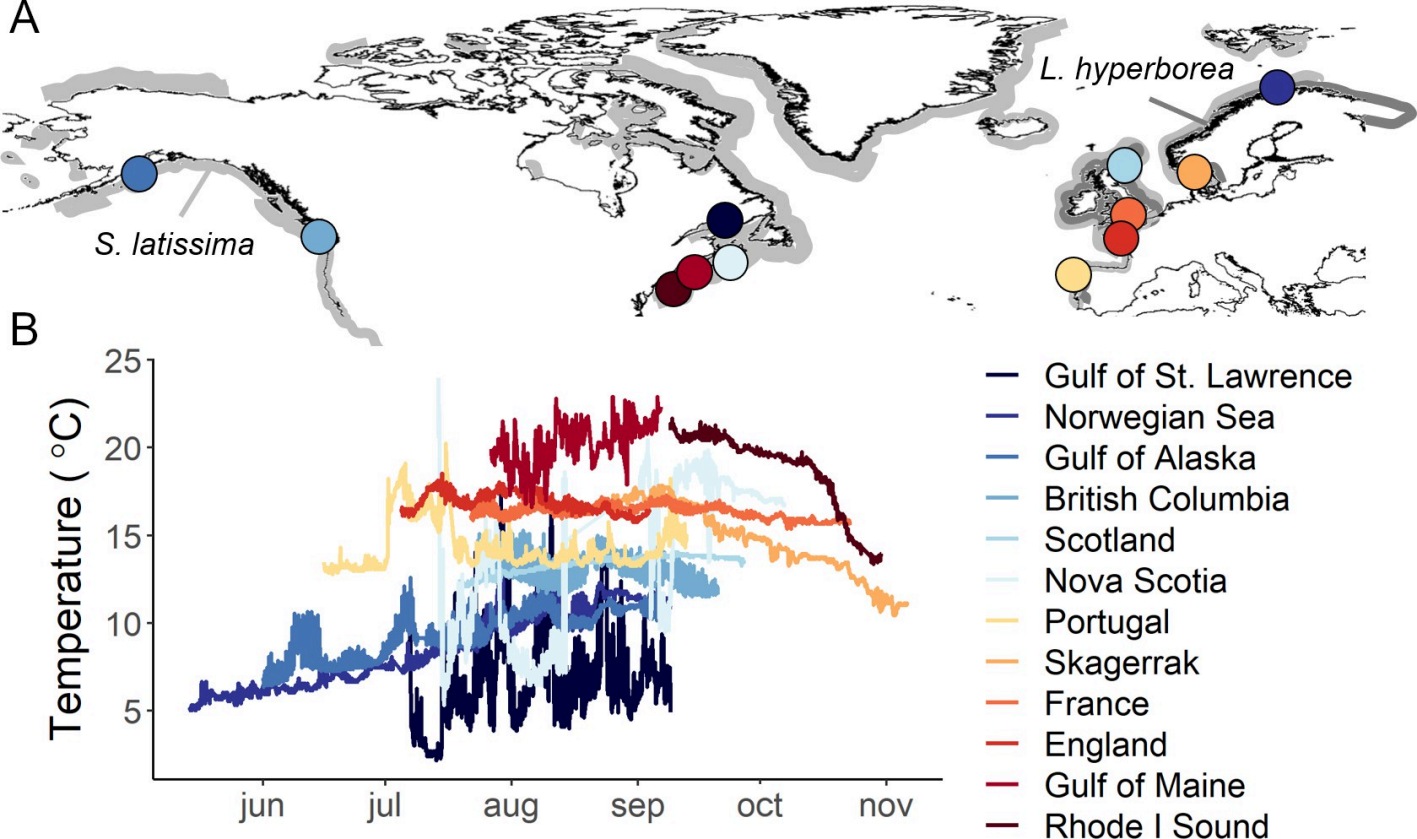
Study regions and ocean temperatures during the experiments. Map of study regions (A) and sea floor temperature records (B) over the duration of the experiment (Data A in S1 Data). Distributions of *Saccharina latissima* and *Laminaria hyperborea* kelps, modified from [41] using map from [42], are shown in light and dark gray, respectively.

Here, we conducted a broadly distributed field experiment at 35 sites spanning 12 geographic regions across the northern hemisphere (Fig 1) to measure in situ decomposition rates and changes in carbon and nitrogen tissue content of kelp detritus in coastal habitats and to assess the influence of an ocean-climate gradient on decomposition. Experiments on 2 dominant species of kelp (*Laminaria hyperborea* and *Saccharina latissima*) were deployed through a collaborative network of researchers in the northeast Pacific Ocean (*n =* 1), the subarctic Norwegian Sea (*n* = 1), the Gulf of Alaska (*n* = 1), the northeast Atlantic Ocean (*n* = 4), and the northwest Atlantic Ocean (*n* = 5). Our study sites spanned 28° of latitude, 169° of longitude, and encompassed the entire distribution of the 2 kelp species and a gradient in mean sea temperature of approximately 14°C. We hypothesized that the large spatial range in environmental conditions would drive significant differences in kelp decomposition rates and that turnover would be faster in areas with warmer temperature, lower light, and higher water movement. Additionally, we used a highly standardized cellulose decomposition assay at all sites to compare decomposition rates between kelp forests and other aquatic ecosystems.

## Results

Our study regions experienced markedly different temperature conditions, with average temperatures ranging from 6 to 21°C and regional minimum and maximum temperatures spanning from 2 to 24°C, over the 55- to 121-day deployments (Fig 1 and S1 Table).

**Fig 2:**
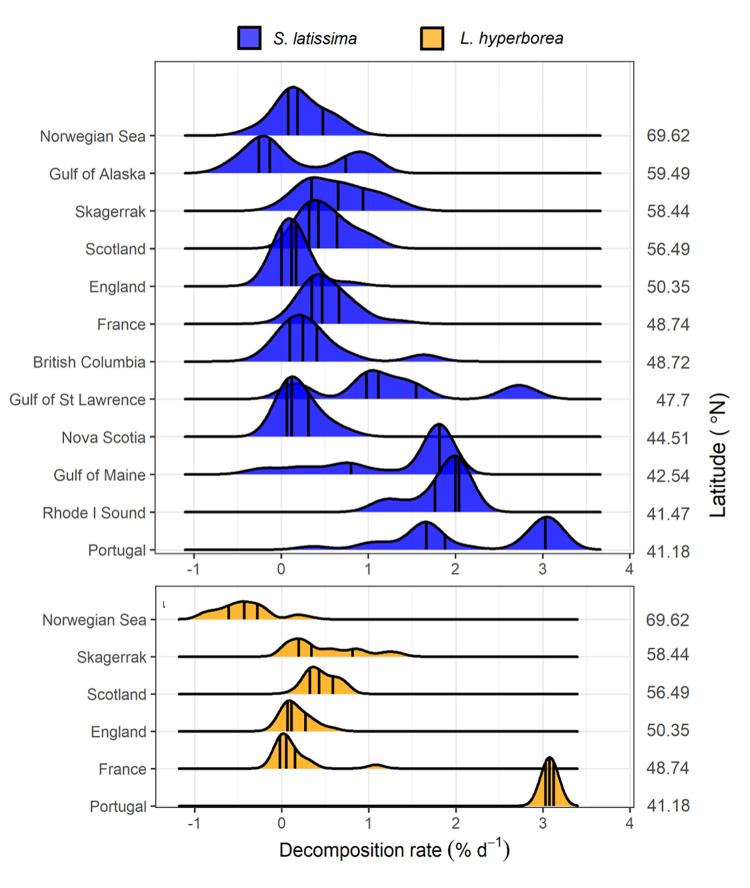
Kelp decomposition rates across study regions. Probability density functions of decomposition rates of (A) *Saccharina latissima* and (B) *Laminaria hyperborea* throughout the northern hemisphere (Data B in S1 Data). Curves show frequency of observations, pooled across sites in each region and ordered by latitude. Black middle lines show medians, and outer lines show the 25th and 75th quantiles. Y axes units are the proportion of observations, ranging from 0 to 1, with the height of each site panel showing 0 to 0.18 (A) and 0 to 0.9 (B).

Across all our study regions, kelp biomass decomposed at an average rate of 0.74 ± 0.87% d^−1^ (± SD) reaching 50% loss after 67 days, on average. Decomposition rates for both species were inversely related to latitude along the 28° gradient (Fig 2). The most rapid biomass loss occurred at the southernmost sites in Rhode Island Sound, United States of America (1.76 ± 0.39% d^−1^) and Portugal (2.63 ± 0.66% d^−1^). Biomass loss was similar among the Gulf of Alaska and other regions in cooler parts of the northeast Atlantic Ocean, with extremely slow decomposition rates (0 to 0.28% d^−1^) over the 72- to 121-day duration of the experiment, especially in the Norwegian Sea and Gulf of Alaska (Fig 2).

**Fig 3:**
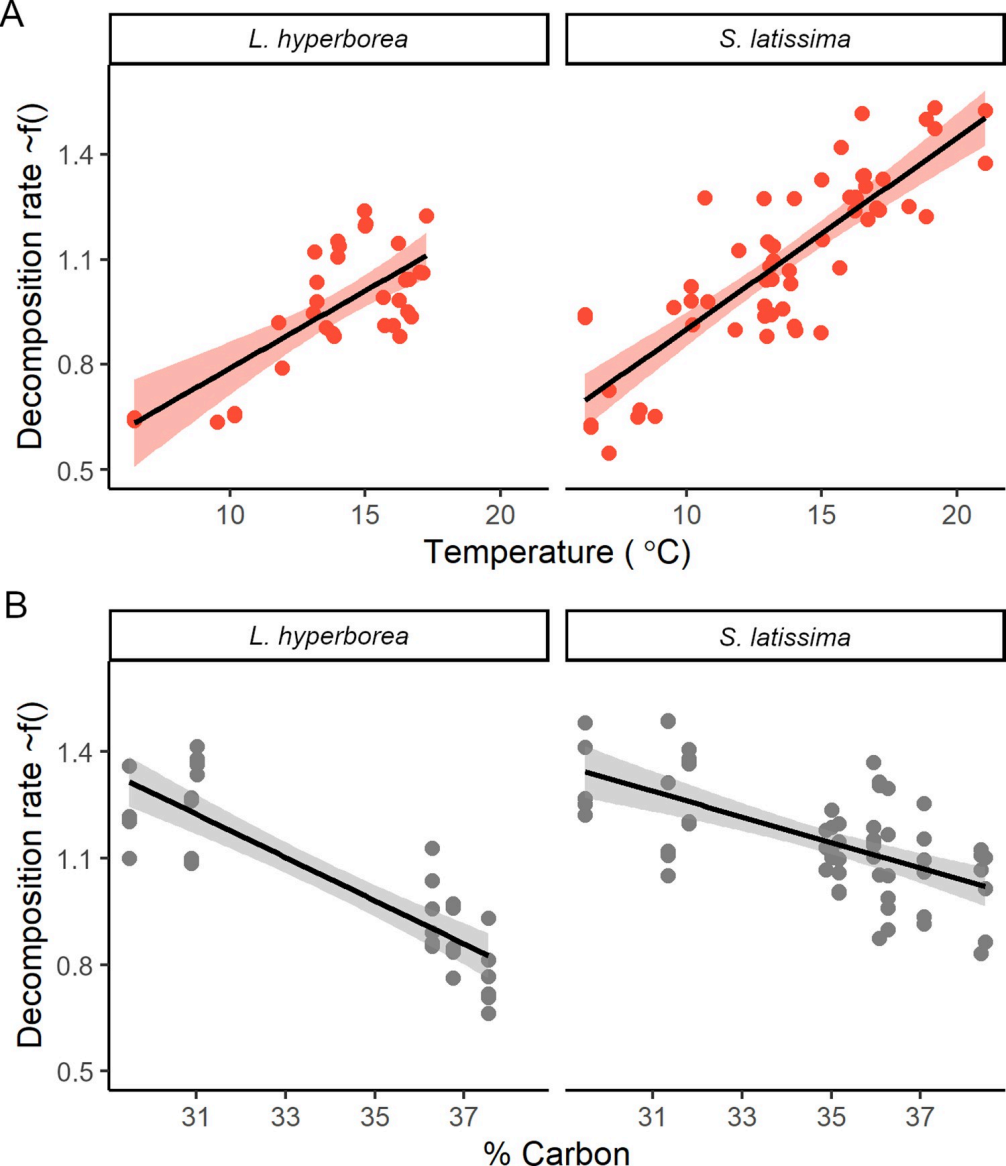
Relationships between decomposition rate and water temperature, carbon content, and species. Relationships between kelp decomposition rate (% d^−1^) and significant predictor variables in generalized linear models: (A) average water temperature during the experiment and (B) initial % carbon content for both species, from the generalized linear mixed effect models, with all other variables in the model held fixed (Data C in S1 Data). Black lines are the expected value from the model, shaded error bar (a and c) is confidence interval, and points are partial residuals for each sampling time at each site. Plots are created with R package visreg [44].

We used generalized linear mixed models to describe relationships between decomposition rates and environmental conditions on the seafloor (water temperature [average and range], light, water movement), as well as algal material traits (species, initial % carbon and % nitrogen), while accounting for study region and site (Tables 1 and S2). These models showed a significant positive relationship between kelp decomposition rate and average sea temperature (Fig 3A), which explained 72% of the variation of all fixed and random effects. There was a negative correlation between average temperature and latitude across our study sites (Pearson’s R = −0.59, *p* < 0.001, *n =* 35), but there was variation around this trend, likely due to the influence of factors independent of latitude on temperature, such as ocean currents (e.g., Gulf Stream and Labrador Currents). We found no evidence that differences in water movement or light intensity influenced kelp decomposition, which we expected would either increase mechanical breakdown or delay tissue necrosis by sustaining low levels of photosynthesis [43]. Average light intensity was highly variable across the study regions (range 6 to 210 Lux), but average water movement was similar (range 1.10 to 1.62 g^3^; S3 Table), possibly due to consistent wave dampening by the cages, which could explain its low importance in the model.

**Table 1:** Generalized linear mixed-effects models. GLMM relating the decomposition (% d^−1^) of kelp detritus to environmental conditions and tissue properties at 12 regions of the northern hemisphere. Temperature (average and range) is temperature at the seafloor over the duration of the experiment. Light is scaled average light (Lux) over the first 2 weeks of the experiment. GLMMs are with gamma distribution and identity link function with predictors temperature (range, average), light and species, initial % carbon and % nitrogen content. Importance of fixed effects parameters were evaluated using likelihood ratio tests with single-term deletions. Shown for each deletion are percentage of deviance explained (% De) and chi-squared statistic used to compare model with deletion to full model. Site and region represent random effects (*n =* 12 regions).

**Model 1** **Fixed effects**	**Log-likelihood**	**% De**	**Chi-squared**	***p***
All parameters	−8.62			
Average temperature	−12.7	32.5	8.31	**0.004**
Temperature range	−8.64	0.21	0.04	0.845
Light	−8.89	2.86	0.51	0.476
% Nitrogen	−9.46	8.81	1.67	0.197
% Carbon	−11.31	23.7	5.37	**0.021**
Species	−14.24	39.4	11.2	**0.001**
**Random effects**	**N**	**Variance**	**SD**	
(1 | Site:Region)	34	0.017	0.130	
(1 | Region)	12	0.155	0.393	
Residual		0.031	0.177	

**Fig 4:**
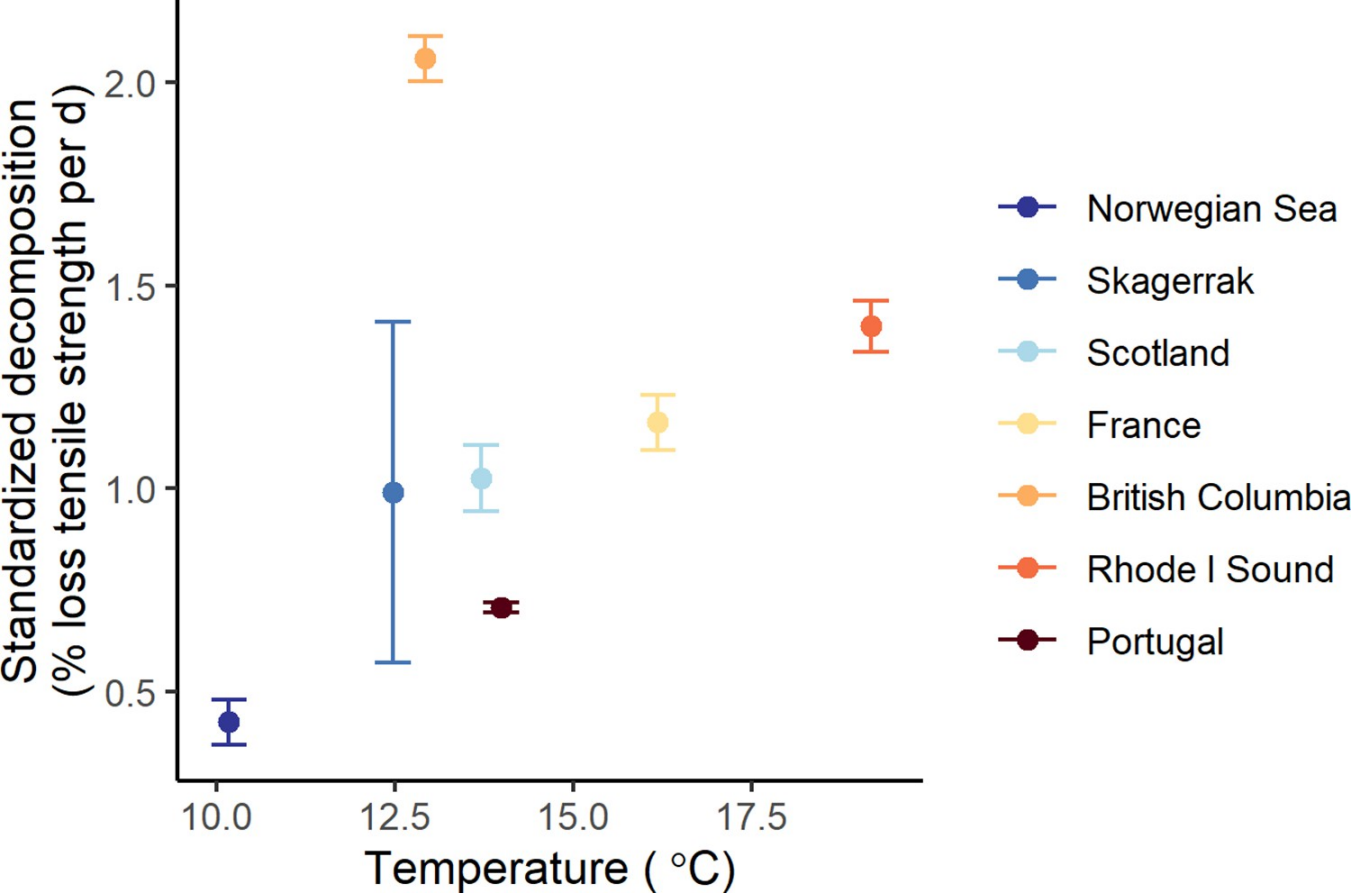
Carbon processing capacity at study sites. Relationship between the carbon processing capacity of the temperate reef ecosystem and temperature on the seafloor over the deployment period. Decomposition is loss of tensile strength per day of cotton strips at each study region (average and SD over sites) (Data D in S1 Data).

The 2 kelp species had different decomposition rates, with *S*. *latissima* losing biomass significantly faster than *L*. *hyperborea* (Fig 3 and Table 1). Decomposition rates were more variable among regions than among sites within regions suggesting that heterogeneity in local conditions did not overshadow the larger spatial patterns in decomposition (Table 1). Initial % carbon content in detrital tissue had a significant effect on the decomposition rates during the experiment, with slower decomposition rates for detritus with higher carbon content (Table 1 and Fig 3B). The background capacity of the surrounding benthic environment at the different study sites to breakdown organic material was assessed using standardized cotton (cellulose) strip assays in 7 of the 12 regions [45] and revealed a positive but not significant relationship with decomposition and mean seafloor temperature during the deployment of the cotton strips (Pearson’s r = 0.38, *p* = 0.4005; Fig 4). The lack of statistical significance was primarily driven by 1 outlier location (British Columbia; without this location the correlation was significant, r = 0.89, *p* = 0.0189). The assays showed an approximately 0% to 2% loss of tensile strength per day (average 0.9 ± 0.55 SD) for our reef sites, which is about half the breakdown rate of this same assay in freshwater streams (1.7 ± 0.83 SD) [45].

**Fig 5:**
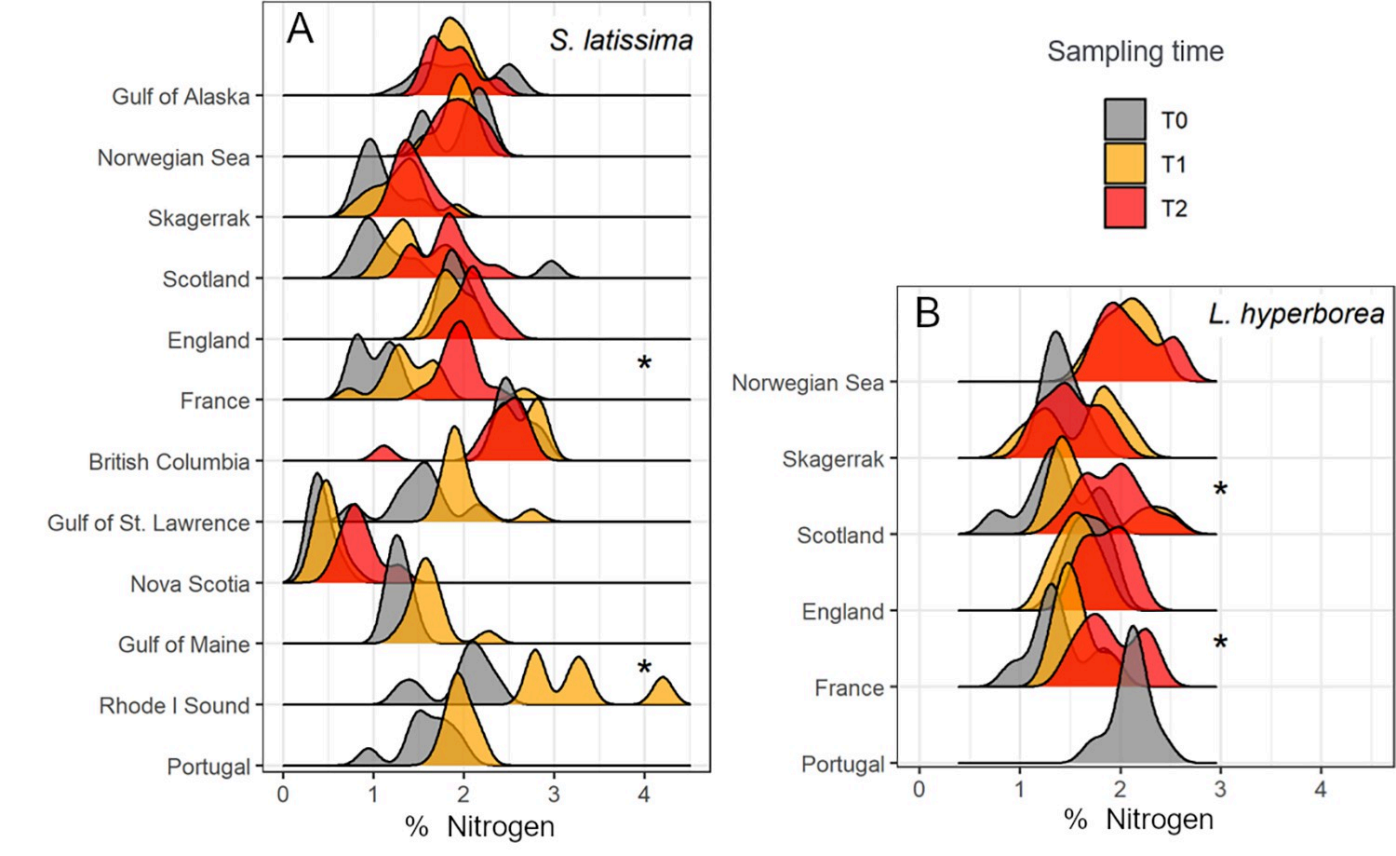
Change in detritus quality with decomposition. Total nitrogen content in kelp detritus over the experiment for *Saccharina latissima* and *Laminaria hyperborea*. Data are frequency measures of % nitrogen from tissue samples taken at the onset of the experiment (T0), the first sampling time (T1), and the final sampling (T2). Y axes units are the proportion of observations. Measures are pooled across sites for each region and ordered by decreasing latitude. Values are missing for later samplings in some regions because insufficient biomass remained for analysis at the time of sampling (* denotes statistical significance, post hoc tests in S4 Table, Data B in S1 Data).

Over the experiment, the average nitrogen content increased significantly in *S*. *latissima* and *L*. *hyperborea* detritus in 2 regions (*S*. *latissima*: France and Rhode I Sound; *L*. *hyperborea*: France and Scotland), and C:N ratios declined significantly in some regions (*S*. *latissima*: Skagerrak, Scotland, France, and Nova Scotia; *L*. *hyperborea*: Scotland and France), suggesting that these kelp tissues became nitrogen enriched as they underwent degradation (Fig 5). We did not detect a relationship between changes in kelp tissue composition (% nitrogen or C:N) and temperature, light, or water movement over the course of the experiment (S3 Table). Isotopic values of kelp detritus did not change over the experiment, apart from in the Norwegian Sea where δ^15^N in *S*. *latissima* and *L*. *hyperborea* increased between initial and final sampling times (S1 and S2 Figs). The % nitrogen in kelp tissue at the onset of the study was highly variable among regions (Fig 5), which likely reflects different background nutrient levels or initial kelp condition, but this variable did not influence decomposition rates (Table 1).

**Fig 6:**
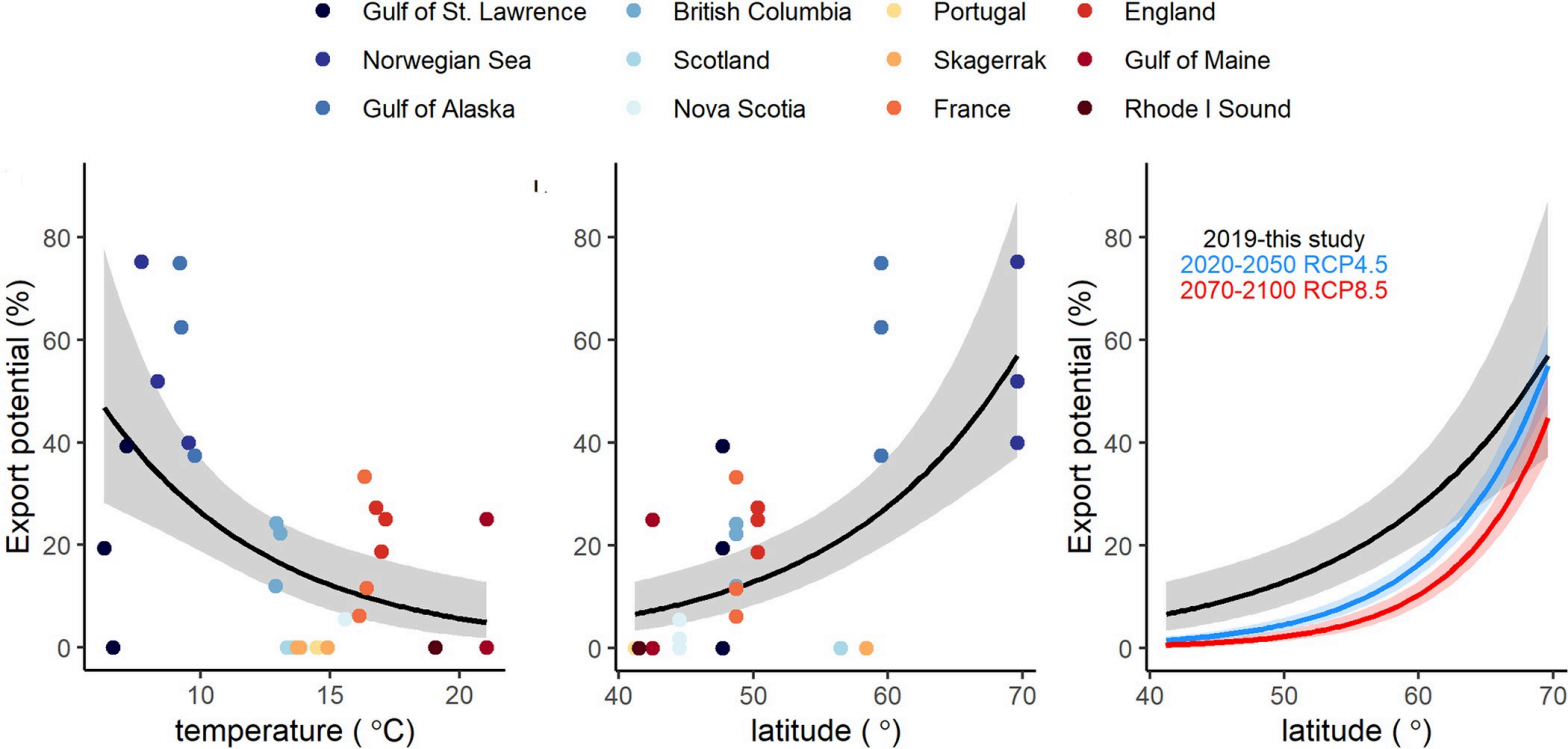
Export potential of kelp carbon with temperature and latitude. Relationships between export potential of kelp material to the deep ocean and (A) temperature at the sea floor and (B) latitude at our study sites. Export potential represents the percent of detrital material that could cross the shelf break (200 m isobath) and sink to the deep sea, which was calculated using decomposition rates and average coastal residence times (days) simulated for each site location [46] (S4 Fig). (C) Predicted changes in export based on predicted sea surface temperature increase under short-term (2020–2050) RCP4.5 and long-term (2070–2100) RCP8.5 scenario in the north polar and northern subtropical regions [50]. Colors (A, B) show regions and fitted lines shows generalized linear model with log link function, with 95% confidence interval shaded (Data E in S1 Data).

The estimated export potential of kelp carbon to the deep ocean, calculated using modeled coastal residence times (CRTs) for our study sites [46] combined with our measured decomposition rates, was greater for sites at higher latitudes and was negatively related to temperature at the sea floor (Fig 6). High variation around these relationships were partly due to variation in modeled simulations of CRTs (the time for exchange between coastal waters and open ocean waters) at our study sites. Transport dynamics of detritus can be complex, and this model is therefore a coarse tool for understanding export potential. However, large amounts of kelp and other phytodetritus are passively transported in the water column with ocean currents or as bedload along the seafloor, making movement of coastal water a useful start for understanding export potential [40,47–49]. Based on the relationship between temperature and export shown in this study, a sea temperature increase of 0.4°C (as projected by RCP 4.5 for 2020 to 2050) would mean an average of 1.4% less of the total detrital kelp production reaching deep ocean sinks, or a 9% decrease in carbon sequestration potential. For a 1.4°C (RCP 4.5 for 2070 to 2100), this becomes 4.1% less export, or a 26% decrease in carbon sequestration potential, and for a 2.7°C increase (RCP 8.5 for 2070 to 2100), this becomes 6.7% less export, or a 43% decrease in carbon sequestration potential (Fig 6).

## Discussion

Our experiments revealed significant variation in the capacity of coastal ecosystems to decompose kelp carbon across broad spatial scales. This was primarily attributed to differences in temperature at study sites across the northern hemisphere, with slower decomposition in cooler northern regions relative to warmer southern regions. This pattern was similar to the standardized carbon processing assays, which suggested increasing capacity of the surrounding ecosystem to break down organic material at lower latitude sites with higher sea temperatures. Kelp decomposition was also related to species and initial carbon content of detrital tissue, but unlike temperature, the initial carbon content did not vary predictably with latitude and was variable within regions.

Temperature dependence of organic matter decomposition constitutes an important link between climate change and the global carbon cycle [5], including in the ocean where there are large actively cycling pools of organic matter [51–55]. There is a general understanding that temperature regulates the rate of biogeochemical processes and decomposition rates and carbon turnover are faster at lower latitudes, due to increased microbial activity and metabolic rates of detritivores and herbivores in warmer climates [8,20,56,57]. However, empirical evidence shows that these patterns do not hold in many systems, and such temperature relationships may not be universal [58–61], due to complex biogeochemical and enzymatic reactions in sediments and physiological adaptation and succession in microbial communities [62,63]. Nevertheless, these relationships have important implications for potential positive feedbacks of climate change, and they underpin predictions of increased permafrost decomposition from microbial activity [7] and faster soil degradation from increased decomposer activity in some terrestrial regions [6,64] with global warming. The present study shows that such a relationship exists for kelp detritus on a large spatial scale when it decomposes on top of seafloor sediments. Our study also identifies cool regions as possible hotspots for kelp carbon storage and sequestration by providing evidence that kelp detritus in these regions remains intact for longer, increasing its potential for dispersal to deeper, offshore carbon sinks [48].

Importantly, although decomposition varied across regions, kelp detritus decomposed slower than many other dominant sources of organic carbon in the ocean (e.g., zooplankton casings, feces and debris, phytodetritus, bacteria) and at rates similar to other forms of benthic vegetation (e.g., seagrass and other seaweeds) (S3 Fig). This could be related to the physicochemical properties of kelp material, such as the presence of structural compounds and phenols [65]. Also, it could be because the material, even as detritus, can remain viable and photosynthetically active for extended periods in shallow subtidal areas with sufficient light to sustain positive photosynthesis [43]. Slow decomposition may be further accentuated as kelp moves out of shallow coastal waters into cooler deep waters. However, despite slow decomposition in shallow subtidal study areas, we found little support that microbial decomposition ceased before all biomass was lost (i.e., a portion was not bioavailable in the short term), because detrital biomass was lost entirely in some litterbags, particularly at lower latitude sites. This does not account for kelp-derived dissolved organic material (DOM) or particulate organic material (POM), which would have been produced (but not measured) over the experiment, and is thought to consist of both labile fractions that are remineralized in the upper ocean and more refractory fractions [9,10]. Although critical information about transport of detrital POM and DOM to deeper ocean sinks is still lacking in many regions [13,66], timescales of exchanges between coastal waters and deeper ocean (CRTs) can be 10s to 100s of days [46]. Our findings show that kelp detritus can have long enough residence times in the coastal zone to match these timescales and therefore have potential to be transported to deeper regions [40,48]. This is consistent with evidence that a substantial amount of kelp detritus reaches deep marine sinks [13,36,67].

The negative relationship between the initial carbon content in detritus and decomposition rates could indicate that more carbon-rich tissue was less palatable to microorganisms or detritivores. This is supported by other studies showing detritus quality is a key predictor of decomposition [68,69]. The nitrogen enrichment of detritus that occurred in some regions throughout the experiment may be explained by increased microbial colonization, because microflora that colonize the kelp acquire inorganic nitrogen from the environment [70–72]. Yet we found little to no change in isotopic δ^13^C‰ and δ^15^N‰, which can sometimes be altered by microfauna that preferential select δ^15^N on kelp detritus [71]. Carbon content is also influenced by phenology and seasonal growth cycles [43,73,74], and although this was partially controlled for in our experiment by selecting recent tissue, these variables could explain some of the variation in initial %C and %N among regions. Differences in initial %N content may also reflect differences in background nutrients (which can influence decomposition [75]) and can be used to infer available nitrogen in the environment [76]. However, these differences in %N were not related to broader patterns of decomposition. We also detected no relationship between nitrogen enrichment and temperature over the course of the experiment. This finding differs from those of distributed decomposition experiments in freshwater systems that suggest warmer temperature shifts decomposition from detritivore to microbial pathways and increases %N [69].

Unaccounted for variation in decomposition across study sites could reflect differences in many other factors, including physiological adaption of microbial communities or other environmental factors (UV, currents) that are not assessed in this study. We found no relationship between our measures of water movement or light intensity and decomposition, which was contrary to our expectation that water movement would increase mechanical breakdown and biomass loss and that low light intensity would enhance tissue decay. Differences in detritivore pressure could have driven different decomposition rates and may explain the residual variability in the relationships we show. However, detritivore abundances and grazing rates on subtidal rocky reefs are known to be complex, being both patchy in space and species specific [77–79], as well as potentially influenced by many environmental factors, including primary production, upwelling, surface currents, and temperature [80,81]. Furthermore, global meta-analyses of herbivory impacts on primary producers in these habitats show no relationship between both grazer effects and latitude and grazer effects and sea temperature [60]. Consequently, herbivore pressure will most likely not produce a uniform latitudinal pattern in overall decomposition rates, such as that which emerged in this experiment. However, kelp forests with abundant sea urchins will have altered production and export of kelp detritus compared to our measures [40], and consumption of kelp material by sea urchins should reduce its life span in the coastal zone and may alter the transport potential of kelp carbon [48].

Kelp forests are currently changing in distribution and abundance due to climate change [26,29], with implications for the storage and cycling of kelp carbon. *S*. *latissima* and *L*. *hyperborea* are disappearing in parts of their warmer southern range edges [82–84]. Kelp forests in other north Atlantic regions, such as around the British Isles, have undergone structural changes following climate-driven shifts in kelp species distributions [85], also leading to concomitant shifts in rates and timings of carbon fixation and release [86]. Along the west coast of North America, loss of predators and marine heatwaves are driving shifts from kelp forests to sea urchins barrens in some areas [87–89]. The temperature-dependent rates of kelp decomposition uncovered here suggest an overall increase in rates of kelp carbon decomposition as oceans warm. Faster turnover means that detritus will have shorter residence time and lower potential to be transported to the deeper ocean or sequestered by burial in shallow soft sediments [9,48]. This would mean a loss of potential carbon sequestration within the current distribution of kelp forests (e.g., [90]) under future warming. For example, if we apply this to kelp forests in Norway and the Canadian Arctic where maps of extent and total NPP exist (total NPP = 1.09 to 4.3 Tg C y^−1^ [91] and 2.2 to 6.4 Tg C y^−1^ and 10.4 to 30.6 Tg C y^−1^ [92], respectively), a 6.7% reduction of kelp export (long-term RCP8.5 scenario) is equivalent to a loss of 73 to 288 Gg C y^−1^ in Norway and 0.3 to 1.8 Tg C y^−1^ in the Eastern Canadian Arctic. Faster decomposition would also alter the nature of kelp as a resource subsidy, which will have ramifications for detrital food webs within kelp forests and in adjacent habitats that rely on this source of production [25].

However, the predicted expansion of kelp forests along Arctic coasts due to reduced sea ice [93] could lead to larger and more productive kelp forests in cooler regions, where decomposition rates appear slower and long-term carbon sequestration more likely [32,93,94]. The consistent changes in decomposition across latitudes highlights the issues with representing major processes underpinning carbon cycling in the ocean in a uniform manner across space. While these patterns should be better understood, incorporating them into estimates of carbon transport in a future ocean will improve current predictions and better resolve the climate mitigation potential of kelp forests. Indeed, key processes such as decomposition at the ecosystem level should be explored further and eventually lead to a fuller understanding of carbon cycling on a global scale.

## Materials and methods

Fieldwork and laboratory analyses were conducted by a collaborative network covering the global range of 2 dominant and broadly distributed kelp species (*S*. *latissima* and *L*. *hyperborea*) (Fig 1). Field decomposition rates of kelp detritus were quantified in concurrent, standardized litterbag experiments deployed in 12 regions throughout the northern hemisphere. Litterbag experiments are widely used to quantify decomposition rates in the field [95] by measuring the mass loss of plant material enclosed in mesh bags that allow water flow and microbial colonization while excluding large grazers and preventing biomass advection. In each region, 3 sites, approximately 0.5 to 10 km apart, were selected. Sites were sand or coarse sediment substrata adjacent to rocky reefs in areas with low to moderate wave and current exposure (S1 Table). Litterbags were preassembled and shipped to all partners, ensuring identical treatments were deployed in all regions. We targeted overall patterns of kelp loss rather than attempting to distinguish between mesograzers (or detritivores) and microbial activity. Consequently, we did not vary mesh size of the litterbags to restrict detritivores as this can substantially alter light and water flow, which may affect kelp decomposition and alter our ability to detect relationships between decomposition and other environmental variables.

In each of the 12 regions, divers haphazardly collected 34 to 36 adult blades with minimal to no epibionts of each targeted species. Six regions collected and deployed 2 species (*S*. *latissima* and *L*. *hyperborea*), and 6 regions deployed 1 species (*S*. *latissima*) (S1 Table). A subset of these collected kelp samples (*n =* 10 to 12) were dried and used for baseline analyses of carbon and nitrogen content. For the remaining 24 blades, a 20-g piece of kelp tissue was sectioned approximately 15 cm from the base and at least 15 cm from the distal end and weighed to the nearest 0.1 g (average = 19.8 ± 0.16 SE). This approach was chosen to maximize blade uniformity across regions as older distal tissue would be less uniform depending on age and fouling. (Compared to the kelp tissue used in the experiment, older kelp tissue may decompose faster and stipe material or newer blade tissue slower, which could over or underestimate residence times of all available detritus types.) Using newly formed basal tissue also minimized phenological or seasonal differences in detrital material from slight variation in timing of the trials across regions, which may influence the decomposition rates.

A single kelp piece was loosely packed into each litterbag (approximately 1 × 1-cm plastic mesh bags) and placed into cages (4 litterbags in each of the 2 cages for each species at each site). Cages were 20 cm by 20 cm by 40 cm and made of plastic 1 × 1 cm mesh (“gutter guard”). Each cage was tethered with cable ties to a weight on the seafloor at approximately 10-m depth. Cage size was selected to allow access of mesograzers and detritivores, but to exclude grazing by sea urchins in our experiments, which can drive localized increases in the turnover, size, and availability of kelp detritus in some areas [40] and could overwhelm measures of turnover in areas where they were locally abundant. All kelp pieces were kept damp after collection, stored in a dark cooler, and deployed within 24 hours of collection.

Environmental variables known or predicted to influence decomposition were measured concurrently throughout the experiment at each site. Hourly light and temperature were measured by an Onset HOBO pendant temperature and light logger fixed to the top of a cage at each site. Only light records for the first 2 weeks of deployment were used to account for fouling of the sensor, which could shade and confound measurements over time. To estimate wave action, an Onset HOBO G logger was placed inside a mesh bag and added to a cage at each site to log hourly movement of the litterbags. We used the average sum of logged acceleration along 3 axes (x, y, and z, units of g^3^) over the period as a relative measure of movement of the litterbags.

Approximately 4 to 6 weeks into the experiment, half the litterbags were collected (2 from each cage, 4 per site). The remaining litterbags were collected after 12 to 18 weeks. At 5 sites, the litterbags were lost at 1 sampling time (S1 Table). Samples were processed within 10 hours of collection. All kelp fragments were removed from bags, patted dry, and weighed to the nearest 0.01 g. Weighed samples were rinsed in distilled water, oven dried at 60°C for 48 hours, and then shipped to the University of California (Davis, California, USA) where they were analyzed for nitrogen and carbon tissue content as well as δ^13^C‰ and δ^15^N‰.

At 17 sites across 7 regions, a standardized cotton strip assay was deployed to quantify the inherent capacity of the temperate reef ecosystem to process organic carbon [22,45]. This cotton strip assay is sensitive to temperature and nutrient availability [96] and integrates the influence of environmental factors, along with the activity of the microbial community, on controlling organic carbon decomposition. The cotton strips were placed in litter bags between the first and final retrieval (*n* = 2 to 4 per site), which was 3 to 5 weeks and comparable to the amount of time estimated to maximize the sensitivity of the assay to environmental conditions [45]. Upon retrieval, cotton strips were cleaned, dried, and returned to the coordinating laboratory for standardized measurements of decomposition (tensile strength loss, which reflects microbial catabolism of the cellulose material). Tensile loss was divided by number of days deployed in the field, as these assays exhibit linear change in strength over time [45]. The timing of the assay on the first retrieval meant it was not possible to directly associate these organic decomposition rates with kelp decomposition rates; however, it does provide a measure of the relative difference in capacity of the ecosystem to break down carbon across our study regions.

We compared the obtained values of kelp decomposition to that of other marine detritus using data from litterbags or incubations obtained from the literature (S5 Table). Decomposition rates for seaweed, seagrass, mangrove, other particulate detritus were obtained from Web of Science using key words “decay,” “decomposition,” “litter,” “half-life,” “k,” and the habitat names. Decomposition rates for POM and DOM were obtained from global reviews and global models of decay rates of these materials [53,97,98]. For each type of organic material (seaweed, seagrass, mangrove, other particulate detritus (e.g., marine snow, zooplankton feces or debris), and DOM), we calculated residence times (days to 50% loss). This metric enabled comparison between materials with different decay functions.

We estimated export potential of detrital kelp material at each site using global models of CRT by Liu and colleagues [46]. CRT was defined as the elapsed time in days for a parcel of source water in the coastal domain (defined by the 200-m isobath) to exit to the open ocean. The average CRT for each study site was obtained from these models using nearest neighbor analysis on the 0.125° resolution model, which was averaged from 1998 to 2007. We converted CRT to kelp export potential by multiplying average decomposition rate (% loss per day) and average CRT in days at each site, using an upper limit of 100% loss or 0% export potential. To examine how representative site-level estimates of CRT were compared to the CRT for the broader coastal area, we compared these estimates to average residence times for the larger ecoregion that each site occurred in [99] (S4 Fig). Although this CRT model is based on the NOAA Modular Ocean Model, which was the highest available resolution current model that covers all our study regions [100], it still represents a coarse approximation of water movement in the coastal zone, and so only provides a first-order estimate of export potential. Resolving the true export requires improved high-resolution ocean current models for the coastal zone.

### Analysis

Rates of kelp loss (average rate of biomass loss for each retrieval time at each site) as a function of environmental conditions and kelp tissue properties were analyzed by generalized linear mixed effects models. Sites were averaged because litterbags in the same cage were not independent replicates. We also calculated 2 k values, using the equation y = *e*^-kt^ and y = *e*^-kt^ **+ R**, where *y* is the proportion of biomass remaining at a time point, *t* is the time elapsed since the beginning of the experiment (days), and R is the **residual portion of biomass with very little decay on these timescales (estimated as 10% of the initial WW)** (S1 File). We added 1 g to WW in all k calculations because LN(WW_t_ = 0) is undefined. However, linear rates of loss were deemed more appropriate for comparing decomposition among sites and regions for our dataset, because we had only 3 time points (including baseline) for each site, some sites with both 0% and 100% biomass loss, and total experiment length differed among regions. The lagged onset of decomposition and lack of rapid initial biomass loss in some of our study regions further supported the use of linear decomposition rates, which is a similar approach to other regional decomposition experiments on kelp detritus [43,101], although it deviates from patterns of exponential decay shown for other types of organic material [102]. Because we were examining kelp decomposition, any negative rates of loss (biomass increase or growth) were assigned a value of 0 in our model, assuming a growing kelp is undergoing little to no decomposition. This assumption was supported by a lack of significant change in tissue content of these fragments, no visible evidence of senesce, and previous studies showing kelp detritus on the seafloor in subtidal habitats can remain partially viable (e.g., grow, photosynthesize) for weeks [43]. Our predictor variables were obtained from logger data and stable isotope measures. The fixed effects were kelp species, average water temperature at the seafloor, range in water temperature, average light conditions, and relative water movement during the experimental period, as well as site nested within region as the random effects. We used 2 variables to capture temperature conditions, the average temperature over the deployment and the temperature range (the difference between the 10th and 90th percentiles) as temperature ranges varied markedly, from 0.6 to 18.6°C. Average temperatures and peak temperatures (90th percentile) were highly correlated among sites (Pearson’s R = 0.96, *p* < 0.001), so peak temperatures were not included in our model. Temperature loggers were lost in the Gulf of Maine region, so temperatures were obtained from the closest meteorological weather buoy (19 km away).

We accounted for differences in starting kelp conditions using initial % carbon and nitrogen content in kelp tissue as fixed effects in the model. Initial % nitrogen was strongly correlated with initial C:N ratio (Pearson’s correlation tests, R = −0.837, *p* < 0.001), and C:N was therefore not included in the model. Log-likelihood tests using Akaike information criterion (AIC) showed that the model with the variable % nitrogen fit the data slightly better than with C:N (and produced similar results), so we used %N (difference in AIC = 0.88). Water movement was modeled separately using a subset of the data, because these measures were not available for Gulf of Maine and the Gulf of St. Lawrence. The main relationships between the other key variables (light, temperature, species) were similar in both models. To confirm the latitudinal gradient was statistically significant, we ran another model using the continuous variable of “latitude” as a predictor of biomass loss instead of a categorical variable (region name) (S1 File). We did not use “latitude” in our final model because it was correlated with temperature and the environmental gradients underlying these latitudinal differences provided more interesting and operational information on spatial patterns of carbon turnover. Decomposition of organic material from the cotton strip assay were compared across regions using Pearson’s correlation tests between loss of tensile strength per day and average temperature on the seafloor. Latitude and temperature at the seafloor at the assay sites were highly correlated (r = −0.78, *p* = 0.040), so only temperature was analyzed. We tested for significant changes in tissue of *S*. *latissima* and *L*. *hyperborea* using multiple 2-way ANOVAs comparing %N, %C, C:N, δ^13^C‰, and δ^15^N‰ at the start and end of the experiment among regions. Post hoc comparisons were conducted for each region using Tukey’s tests.

All analyses were conducted in R (version 3.5.3). We used the glmer function from package *lme4* to fit the generalized linear mixed-effects models (glmm) and the glm function to fit the generalized linear models (glm) with a log link function for the proportion kelp material exported beyond the continental shelf. Decomposition models were fit with a gamma distribution and identity link function. We used the fitted glmm to predict decomposition rates under 3 different scenarios of warming: sea surface temperature increase under short-term (2020 to 2050) RCP4.5 (+0.46°C) and long-term (2070 to 2100) RCP4.5 (+1.44°C) and RCP8.5 (+2.7°C). We used predicted SST changes for these scenarios for the north polar and northern subtropical regions of the world, which corresponded to our study site locations (calculated using the average of clusters PRN and STRN from [50]). We then used these updated future decomposition rates to calculate potential export under these 3 scenarios, and fit the results to glms with a log link function. We checked all model residuals for violation of model assumptions and to investigate the suitability of the chosen distribution (i.e., deviance residuals versus theoretical quantiles), dispersion, and heteroscedasticity, using package *DHARMa* (S1 File). To stabilize parameter estimation, we standardized mean light by dividing it by 100, so it matched the scale of the other predictor variables. We used likelihood ratio tests with single-term deletions to assess the importance of each fixed effect predictor in the models. Relationships between the most important predictor variables and decomposition rates were illustrated with package *visreg*, which shows the relationship between a single predictor and the model outcome while holding the other predictors constant [44].

## Supporting information

S1 Table Locations of 35 study sites in each region with times of deployment (T0) and retrievals (T1 and T2) (Data B in S1 Data). (DOCX)

S2 Table Summary of generalized linear mixed-effects models (GLMMs) relating the decomposition (% d^−1^) of kelp detritus to environmental conditions and tissue properties at 12 regions of the northern hemisphere. Temperature (average and range) is temperature at the seafloor over the duration of the experiment. Light is average light (Lux) over the first 2 weeks of the experiment. The % carbon is the initial carbon content in the kelp detritus, and water movement is average g forces within the cages over the experiment. GLMMs are with gamma distribution and identity link function. Model 1 uses the full dataset (*n =* 12 regions) with predictors temperature (range, average), light and species, and model 2 uses a subset of the data (*n* = 9 regions) with additional predictors % carbon content and water movement, because these variables were not obtained at all 12 regions. Site and region represent random effects.
(DOCX)

S3 Table ANOVA and *t* tests of nitrogen enrichment (%N content) for kelp species in each region over the duration of experiment. Tukey’s post hoc tests are performed to compare between initial and final sampling time in each region.
(DOCX)

S4 Table Standardized cotton strip assays showing decomposition of cellulose in study sites. Measure is percent loss of tensile strength per day.
(DOCX)

S5 Table Decomposition rates (average ± SD) and residence times reported for different types of marine detritus. (DOCX)

S1 Fig Change in nitrogen isotopic values of kelp detritus. δ^15^N in *S*. *latissima* (A) and *L*. *hyperborea* (B) kelp detritus over the experiment. Data are frequency measures of ‰δ^15^N from tissue samples taken at the onset of the experiment (T0), the first sampling time (T1), and the final sampling (T2). Y axes units are the proportion of observations. Measures are pooled across sites for each region and ordered by decreasing latitude. In some regions, insufficient tissue remained for T2 (Data B in S1 Data).
(DOCX)

S2 Fig Change in carbon isotopic values and carbon content of kelp detritus. δ^13^C in *S*. *latissima* (A) and *L*. *hyperborea* (B) and percent carbon content in *S*. *latissima* (C) and *L*. *hyperborea* (D) kelp detritus over the experiment. Data are frequency measures of ‰δ^13^C and %C from tissue samples taken at the onset of the experiment (T0), the first sampling time (T1), and the final sampling (T2). Y axes units are the proportion of observations. Measures are pooled across sites for each region and ordered by decreasing latitude. In some regions, insufficient tissue remained for T2 (Data B in S1 Data).
(DOCX)

S3 Fig Residence time of marine detritus. Residence times (days to 50% decomposition) reported for different types of marine detritus, including kelps from our study regions (Sl = *Saccharina latissima*; Lh = *Laminaria hyperborea*) and measures reported in the literature for other seaweeds, seagrass, mangrove detritus (leaf), other POM and DOM (S5 Table). POM are from various sources, including zooplankton debris, feces, fauna casings, and marine snow. DOM are labile DOC or DOM released from zooplankton debris or marine snow during incubations. Refractory components of DOC are not included and residence times for these organic carbon pool can range from years to decades or more. DOC, dissolved organic carbon; DOM, dissolved organic material; POM, particulate organic material.
(DOCX)

S4 Fig Coastal residence time. Average coastal residence times (days) simulated for each site location using global models of coastal residence time for water parcels exiting to the open ocean across the 200-m isobath (CRT) by Liu and colleagues (1) using the 0.125° resolution model, which was averaged over 1998–2007. Colors show study regions, ordered from left to right by decreasing latitude. Circles show average (±SE) site-level estimates of CRT. Crosses show average CRT for larger ecoregion (2) that the sites occurred (Data F in S1 Data).
(DOCX)

S1 File Additional model and model fit assessments. (DOCX)

S1 Data Supporting data. File containing sheets for Data A, Data B, Data C, Data D, Data E, Data F, and metadata.
(XLSX)

## Abbreviations

AIC: Akaike information criterion
CRT: coastal residence time
DOM: dissolved organic material
POC: particulate organic carbon
POM: particulate organic material
