# Supplementary material for: Kelp carbon sink potential decreases with warming due to accelerating decomposition
Source: PLoS Biol. 2022 Aug 4;20(8):e3001702. doi: 10.1371/journal.pbio.3001702 (PMC9352061; doi:10.1371/journal.pbio.3001702)
Supplement: S1 Table — (DOCX) [file pbio.3001702.s001.docx]

**S1 Table.** Locations of 35 study sites in each region with times of deployment (T0) and retrievals (T1 and T2).

| **Region** | **Site** | **Latitude** | **Longitude** | **Species** | **T0** | **T1** | **T2** |
| --- | --- | --- | --- | --- | --- | --- | --- |
| Gulf of Alaska | Hesketh | 59,50722 | -151,54919 |  | 30-May-18 | 9-Jul-18 | 6-Sep-18 |
|  | Jakolof | 59,46843 | -151,53777 | *S. latissima* | 30-May-18 | 9-Jul-18 | 6-Sep-18 |
|  | Herring | 59,48115 | -151,52243 |  | 30-May-18 | 9-Jul-18 | 6-Sep-18 |
| British Columbia | Fulford | 48,74803 | -123,43338 |  | 22-Jul-18 | 20-Aug-18 | 21-Sep-18 |
|  | Portland | 48,73232 | -123,37640 | *S. latissima* | 22-Jul-18 | 20-Aug-18 | 21-Sep-18 |
|  | Russell | 48,75055 | -123,40543 |  | 22-Jul-18 | 20-Aug-18 | 21-Sep-18 |
| Gulf of St. Lawrence | Site 1 | 47,70000 | -70,40000 |  | 5-Jul-18 | 9-Aug-18 | 9-Sep-18 |
|  | Site 2 | 47,70000 | -70,40000 | *S. latissima* | 5-Jul-18 | 9-Aug-18 | 9-Sep-18 |
|  | Site 3 | 47,70000 | -70,40000 |  | 5-Jul-18 | 9-Aug-18 | 9-Sep-18 |
| Nova Scotia | Paddy's Head | 44,52720 | -63,95230 | *S. latissima* | 13-Jul-18 | 2-Sep-18 | lost |
|  | Sandy Cove | 44,46197 | -63,70975 | *L. digitata* | 13-Jul-18 | 31-Aug-18 | 7-Oct-18 |
|  | The Lodge | 44,55629 | -64,06705 |  | 13-Jul-18 | 2-Sep-18 | 10-Oct-18 |
| Gulf of Maine | Baker's Island 1 | 42,53698 | -70,79248 |  | 27-Jul-18 | 20-Sep-18 | lost |
|  | Baker's Island 2 | 42,53413 | -70,79403 | *S. latissima* | 27-Jul-18 | 20-Sep-18 | lost |
|  | Baker's Island 3 | 42,53553 | -70,79445 |  | 3-Aug-18 | 20-Sep-18 | lost |
| Rhode I Sound | Fort Wetherill | 41,47732 | -71,39276 | *S. latissima* | 7-Sep-18 | 26-Oct-18 | 14-Dec-18 |
|  | Kings Beach | 41,45294 | -71,34464 |  | 7-Sep-18 | 26-Oct-18 | 14-Dec-18 |
| Skagerrak | S13 | 58,41700 | 8,76258 | *S. latissima* | 24-Aug-18 | 19-Oct-18 | 6-Nov-18 |
|  | S3 | 58,39596 | 8,73951 | *L. hyperborea* | 25-Aug-18 | 18-Oct-18 | 6-Nov-18 |
|  | S5 | 58,50281 | 8,88807 |  | 25-Aug-18 | 19-Oct-18 | 8-Nov-18 |
| Norwegian Sea | Edoya | 69,60939 | 17,90804 | *S. latissima* | 12-May-18 | lost | 10-Sep-18 |
|  | Lost chain | 69,61261 | 17,91701 | *L. hyperborea* | 12-May-18 | 3-Jul-18 | 10-Sep-18 |
|  | Morten's site | 69,63068 | 17,94419 |  | 12-May-18 | 3-Jul-18 | 10-Sep-18 |
| England | Batton Bay | 50,35472 | -4,14960 | *S. latissima*  *L. hyperborea* | 3-Jul-18 | 31-Jul-18 | 4-Sep-18 |
|  | Ramscliff Point | 50,36082 | -4,12942 |  | 3-Jul-18 | 31-Jul-18 | 4-Sep-18 |
|  | Breakwater | 50,33415 | -4,14538 |  | 3-Jul-18 | 31-Jul-18 | 4-Sep-18 |
| Scotland | Danger Reef | 56,47202 | -5,46467 | *S. latissima* | 17-Jul-18 | 29-Aug-18 | 27-Sep-18 |
|  | The Greggs | 56,47843 | -5,51168 | *L. hyperborea* | 17-Jul-18 | 29-Aug-18 | 27-Sep-18 |
|  | Goat Island | 56,52522 | -5,46093 |  | 17-Jul-18 | 29-Aug-18 | 27-Sep-18 |
| France | Roscoff 1 | 48,70889 | -3,92694 | *S. latissima*  *L. hyperborea* | 20-Jul-18 | 20-Aug-18 | 23-Oct-18 |
|  | Roscoff 2 | 48,70917 | -3,95333 |  | 20-Jul-18 | 20-Aug-18 | 23-Oct-18 |
|  | Roscoff 3 | 48,73250 | -3,97139 |  | 20-Jul-18 | 20-Aug-18 | 23-Oct-18 |
| Portugal | Matosinhos N | 41,17738 | -8,70542 | *S. latissima* | 14-Jun-18 | 16-Jul-18 | 13-Sep-18 |
|  | Matosinhos C | 41,17625 | -8,70268 | *L. hyperborea* | 14-Jun-18 | 16-Jul-18 | 13-Sep-18 |
|  | Matosinhos E | 41,17715 | -8,70029 |  | 14-Jun-18 | 16-Jul-18 | 13-Sep-18 |
