# Supplementary material for: Kelp carbon sink potential decreases with warming due to accelerating decomposition
Source: PLoS Biol. 2022 Aug 4;20(8):e3001702. doi: 10.1371/journal.pbio.3001702 (PMC9352061; doi:10.1371/journal.pbio.3001702)
Supplement: S2 Table — Temperature (average and range) is temperature at the seafloor over the duration of the experiment. Light is average light (Lux) over the first 2 weeks of the experiment. The % carbon is the initial carbon content in the kelp detritus, and water movement is average g forces within the cages over the experiment. GLMMs are with gamma distribution and identity link function. Model 1 uses the full dataset (n = 12 regions) with predictors temperature (range, average), light and species, and model 2 uses a subset of the data (n = 9 regions) with additional predictors % carbon content and water movement, because these variables were not obtained at all 12 regions. Site and region represent random effects. (DOCX) [file pbio.3001702.s002.docx]

**S2 Table.** Summary of generalized linear mixed-effects models (GLMM) relating the decomposition (% d-1) of kelp detritus to environmental conditions and tissue properties at 12 regions of the northern hemisphere. Temperature (average and range) is temperature at the seafloor over the duration of the experiment. Light is average light (Lux) over the first 2 weeks of the experiment. The % carbon is the initial carbon content in the kelp detritus, and water movement is average g forces within the cages over the experiment. GLMMs are with gamma distribution and identity link function. Model 1 uses the full dataset (n = 12 regions) with predictors temperature (range, average), light and species, and model 2 uses a subset of the data (n = 9 regions) with additional predictors % carbon content and water movement, because these variables were not obtained at all 12 regions. Site and region represent random effects.

| **Model 1.** | **Estimate** | **SE** | **t** | **p** |
| --- | --- | --- | --- | --- |
| (Intercept) | 1,307 | 0,400 | 3,271 | **0.001** |
| Average temperature | 0,051 | 0,019 | 2,753 | **0.006** |
| Temperature range | -0,003 | 0,019 | -0,142 | 0.887 |
| Light | -0,014 | 0,024 | -0,577 | 0.564 |
| Species | 0,122 | 0,047 | 2,589 | **0.010** |

| **Model 2.** | | | | | |
| --- | --- | --- | --- | --- | --- |
|  |  | **Estimate** | **SE** | **t** | **p** |
| (Intercept) |  | 2,296 | 1,520 | 1,510 | 0,131 |
| Average temperature |  | 0,058 | 0,018 | 3,196 | **0,001** |
| Temperature range |  | -0,020 | 0,021 | -0,954 | 0,340 |
| % Carbon |  | -0,055 | 0,021 | -2,589 | **0,010** |
| Light |  | -0,015 | 0,023 | -0,661 | 0,509 |
| Water movement |  | 0,136 | 0,041 | 3,277 | **0,001** |
| Species |  | 0,057 | 0,116 | 0,488 | 0,626 |
