# Supplementary material for: Kelp carbon sink potential decreases with warming due to accelerating decomposition
Source: PLoS Biol. 2022 Aug 4;20(8):e3001702. doi: 10.1371/journal.pbio.3001702 (PMC9352061; doi:10.1371/journal.pbio.3001702)
Supplement: S3 Table — Tukey’s post hoc tests are performed to compare between initial and final sampling time in each region. (DOCX) [file pbio.3001702.s003.docx]

**S3 Table.** ANOVA and *t*-tests of nitrogen enrichment (%N content) for kelp species in each region over the duration of experiment. Tukey’s post-hoc tests are performed to compare between initial and final sampling time in each region.

| ***S. latissima*** |  |  |  |  |  |
| --- | --- | --- | --- | --- | --- |
|  | **Df** | **SS** | **MSS** | **F** | **p** |
| Region | 11 | 79,41 | 7,22 | 79,40 | **<0.001** |
| Time | 2 | 6,58 | 3,29 | 36,16 | **<0.001** |
| Region x Time | 20 | 14,94 | 0,75 | 8,22 | **<0.001** |
| Residuals | 285 | 25,91 | 0,09 |  |  |
|  |  |  |  |  |  |
|  |  |  |  |  |  |
| **Tukey's results** |  |  |  |  |  |
| **Region** | **Comparison** | **Est** | **P** |  |  |
| Norwegian Sea | T2,T0 | -0.004 | 1 |  |  |
| Skagerrak | T2,T0 | 0.376 | 0.655 |  |  |
| Scotland | T2,T0 | 0.517 | 0.074 |  |  |
| England | T2,T0 | 0.198 | 1 |  |  |
| France | T2,T0 | 0.942 | **<0.001** |  |  |
| British Columbia | T2,T0 | -0.235 | 1 |  |  |
| Gulf of St. Lawrence | T2,T0 | 0.008 | 1 |  |  |
| Nova Scotia | T2,T0 | 0.437 | 0.635 |  |  |
| Gulf of Maine | T1,T0 | 0.349 | 0.856 |  |  |
| Rhode I Sound | T1:T0 | 1.300 | **<0.001** |  |  |
| Portugal | T1:T0 | 0.382 | 0.829 |  |  |

| ***L. hyperborea*** | |  |  |  |  |
| --- | --- | --- | --- | --- | --- |
|  | **Df** | **SS** | **MS** | **F** | **p** |
| Region | 5 | 5.23 | 1.05 | 13.07 | **<0.001** |
| Time | 2 | 2.50 | 1.25 | 15.62 | **<0.001** |
| Region x Time | 8 | 1.46 | 0.18 | 2.27 | **0.026** |
| Residuals | 143 | 11.44 | 0.08 |  |  |
|  |  |  |  |  |  |
| **Tukey's results** | |  |  |  |  |
| **Region** | **Comparison** | **Est** | **P** |  |  |
| Norwegian Sea | T2,T0 | 0.472 | 0.747 |  |  |
| Skagerrak | T2,T0 | 0.090 | 0.998 |  |  |
| Scotland | T2,T0 | 0.551 | **0.004** |  |  |
| England | T2,T0 | 0.181 | 0.995 |  |  |
| France | T2,T0 | 0.507 | 0.015 |  |  |
