# Supplementary material for: Kelp carbon sink potential decreases with warming due to accelerating decomposition
Source: PLoS Biol. 2022 Aug 4;20(8):e3001702. doi: 10.1371/journal.pbio.3001702 (PMC9352061; doi:10.1371/journal.pbio.3001702)
Supplement: S4 Table — Measure is percent loss of tensile strength per day. (DOCX) [file pbio.3001702.s004.docx]

**S4 Table.** Standardized cotton strip assays showing decomposition of cellulose in study sites. Measure is percent loss of tensile strength per day.

| **Region** | **Site** | **Lat** | **Lon** | **T0** | **T1** | **Tensile strength**  **(% d^-1^)** | **N** |
| --- | --- | --- | --- | --- | --- | --- | --- |
| British Columbia | Fulford | 48,74803 | -123,43338 | 20-Aug-18 | 21-Sep-18 | 2,05 | 5 |
|  | Russell | 48,75055 | -123,40543 | 20-Aug-18 | 21-Sep-18 | 2,08 | 3 |
| Rhode I Sound | Fort Wetherill | 41,47732 | -71,39276 | 26-Oct-18 | 14-Dec-18 | 1,40 | 4 |
|  |  |  |  |  |  |  |  |
| Skagerrak | S13 | 58,41700 | 8,76258 | 19-Oct-18 | 6-Nov-18 | -0,04 | 3 |
|  | S3 | 58,39596 | 8,73951 | 18-Oct-18 | 6-Nov-18 | 1,77 | 4 |
| Norwegian Sea | Lost chain | 69,61261 | 17,91701 | 3-Jul-18 | 10-Sep-18 | 0,37 | 1 |
|  | Morten's site | 69,63068 | 17,94419 | 3-Jul-18 | 10-Sep-18 | 0,45 | 2 |
| Scotland | Danger Reef | 56,47202 | -5,46467 | 29-Aug-18 | 27-Sep-18 | 0,78 | 3 |
|  | The Greggs | 56,47843 | -5,51168 | 29-Aug-18 | 27-Sep-18 | 1,15 | 2 |
|  | Goat Island | 56,52522 | -5,46093 | 29-Aug-18 | 27-Sep-18 | 1,19 | 3 |
| France | Roscoff 1 | 48,70889 | -3,92694 | 20-Aug-18 | 23-Oct-18 | 1,18 | 4 |
|  | Roscoff 2 | 48,70917 | -3,95333 | 20-Aug-18 | 23-Oct-18 | 1,40 | 4 |
|  | Roscoff 3 | 48,73250 | -3,97139 | 20-Aug-18 | 23-Oct-18 | 0,91 | 4 |
| Portugal | Matosinhos N | 41,17738 | -8,70542 | 16-Jul-18 | 13-Sep-18 | 0,73 | 4 |
|  | Matosinhos C | 41,17625 | -8,70268 | 16-Jul-18 | 13-Sep-18 | 0,69 | 4 |
|  | Matosinhos E | 41,17715 | -8,70029 | 16-Jul-18 | 13-Sep-18 | 0,70 | 4 |
