# Supplementary material for: Kelp carbon sink potential decreases with warming due to accelerating decomposition
Source: PLoS Biol. 2022 Aug 4;20(8):e3001702. doi: 10.1371/journal.pbio.3001702 (PMC9352061; doi:10.1371/journal.pbio.3001702)
Supplement: S5 Table — (DOCX) [file pbio.3001702.s005.docx]

**S5 Table**. Decomposition rates (average ± SD) and residence times reported for different types of marine detritus.

| **Organic carbon sources** | **Decomposition**  **% d^-1^** | **Residence time**  **d to 50%** | **Source** |
| --- | --- | --- | --- |
| *S. latissima* | 0.74 ± 0.71^\|\|^ | 68^\|\|^ | This study |
| *L. hyperborea* | 0.76 ± 1.1 | 66 | This study |
| Seagrass (*Posidonia*) | 0.58 ± 0.13 | 88 | (1) |
| Seagrass (*Thalassia*) | 1.6 | 57 | (2) |
| Seagrass (*Haladule*) | 0.37 ± 0.14 | 136 | (3) |
| Seagrass (*Halophila*) | 5.1 | 9.7 | (4) |
| Seagrass (*Cymodocea*) | 0.41 | 120 | (5) |
| Seaweed (*Caulerpa*) | 7.1 | 7 | (6) |
| Seaweed (*Fucus*) | 3.5 | 14 | (7) |
| Seaweed (*Piliayella*) | 1.5 | 34.3 | (8) |
| Seaweed (*Cladophora*) | 1.4 | 35.7 | (8) |
| Seaweed (*Ulva*) | 0.4 ± 0.1 | 125 | (9) |
| Mangrove (*Avcennia*) |  | 15 | (10) |
| Mangrove (*Rhizophora*) |  | 57 | (10) |
| Mangrove (*Ceriops*) |  | 77 | (10) |
| POM (marine snow)^*X^ | 11.9 ± 1 | 4 ± 1 | (11) |
| POM (diatoms & flagellates) | 10 | 5 | (12) |
| POM (ascidian) |  | 3.5 | (13) |
| POM (*Acartia tonsa* carcasses) | 3.9 | 12.9 | (14) |
| POM (*Acartia tonsa* feces) | 1.4 | 34.7 | (14) |
| POM (copepod feces) | 30.2 | 1.7 | (15) |
| POM (zooplankton feces)^¥^ | 19 | 2.67 | (16) |
| DOM (marine snow) | 25 ± 4.3 | 2 | (13) |
| DOM (bacteria) ^X^ | 2.8 | 18 | (17) |
| DOM (diatoms refractory) | 0.08 ± 0.007 | 606 | (18) |
| DOM (diatoms labile) | 1.6 ± 0.6 | 30 | (18) |
| DOM (labile DOC) |  | 10 ± 15 | (19) |
| DOM (labile DOC) |  | 1.0 ± 1.5 | (19) |
| DOM (DOC)^#^ | 0.12 | 387 | (20) |

^||^ averaged across regions. ^*^ Aggregates that include bacteria, plankton, flagellates, and detritus. ^¥^Fecal pellets of *Acartia*, *Evadne*, *Podon*, and calanoid copepods. ^#^50 m depth collection. ^X^ Surface ocean.

**References**

1. M. A. Mateo, J. Romero, Evaluating seagrass leaf litter decomposition: An experimental comparison between litter-bag and oxygen-uptake methods. *J. Exp. Mar. Bio. Ecol.* **202**, 97–106 (1996).

2. P. Rublee, M. Roman, Decomposition of turtlegrass (*Thalassia testudznum konig* ) in flowing sea-water tanks and litterbags: compositional changes and comparison with natural particulate matter. *J. Exp. Mar. Bio. Ecol.* **58**, 47–58 (1982).

3. S. Opsahl, R. Benner, Decomposition of senescent blades of the seagrass *Halodule wrightii* in a subtropical lagoon. *Mar. Ecol. Prog. Ser.* **94**, 191–205 (1993).

4. M. Josselyn, M. Fonseca, T. Niesen, R. Larson, Biomass, production and decomposition of a deep water seagrass, Halophila decipiens ostenf. *Aquat. Bot.* **25**, 47–61 (1986).

5. P. Peduzzi, G. J. Herndl, Decomposition and significance of seagrass leaf litter (*Cymodocea nodosa*) for the microbial food web in coastal waters (Gulf of Trieste, Northern Adriatic Sea). *Mar. Ecol. Prog. Ser.* **71**, 163–174 (1991).

6. S. L. Williams, Decomposition of the tropical macroalga *Caulerpa cupressoides* (West) C. Agardh: Field and laboratory studies. *J. Exp. Mar. Bio. Ecol.* **80**, 109–124 (1984).

7. R. D. Hunter, Changes in carbon and nitrogen content during decomposition of three macrophytes in freshwater and marine environments. *Hydrobiologia* **51**, 119–128 (1976).

8. T. Paalme, H. Kukk, J. Kotta, H. Orav, “‘In vitro’ and ‘in situ’ decomposition of nuisance macroalgae Cladophora glomerata and Pilayella littoralis” in *Nutrients and Eutrophication in Estuaries and Coastal Waters*, vol 164, E. Orive, M. Elliott, V. de Jonge, Eds. (Springer, 2002), pp. 469–476.

9. A. Catenazzi, M. A. Donnelly, Role of supratidal invertebrates in the decomposition of beach-cast green algae *Ulva* sp. *Mar. Ecol. Prog. Ser.* **349**, 33–42 (2007).

10. A. I. Robertson, Decomposition of mangrove leaf litter in tropical Australia. *J. Exp. Mar. Bio. Ecol.* **116**, 235–247 (1988).

11. H. Ploug, H. P. Grossart, F. Azam, B. B. Jørgensen, Photosynthesis, respiration, and carbon turnover in sinking marine snow from surface waters of Southern California Bight: Implications for the carbon cycle in the ocean. *Mar. Ecol. Prog. Ser.* **179**, 1–11 (1999).

12. U. Riebesell, The formation of large marine snow and its sustained residence in surface waters. *Limnol. Oceanogr.* **37**, 63–76 (1992).

13. S. A. Goldthwait, C. A. Carlson, G. K. Henderson, A. L. Alldredge, Effects of physical fragmentation on remineralization of marine snow. *Mar. Ecol. Prog. Ser.* **305**, 59–65 (2005).

14. B.-G. Lee, N. S. Fisher, Decomposition and release of elements from zooplankton debris. *Mar. Ecol. Prog. Ser.* **88**, 117–128 (1992).

15. J. Urban-Rich, Release of dissolved organic carbon from copepod fecal pellets in the Greenland Sea. *J. Exp. Mar. Bio. Ecol.* **232**, 107–124 (1999).

16. A. B. Bochdansky, G. J. Herndl, Ecology of amorphous aggregations (marine snow) in the Northern Adriatic Sea. V. Role of fecal pellets in marine snow. *Mar. Ecol. Prog. Ser.* **89**, 297–403 (1992).

17. D. L. Kirchman, Y. Suzuki, C. Garside, H. W. Ducklow, High turnover rates of dissolved organic carbon during a spring phytoplankton bloom. *Nature* **352**, 612–614 (1991).

18. B. Fry, C. S. Hopkinson, A. Nolin, B. Norrman, U. L. Zweifel, Long-term decomposition of DOC from experimental diatom blooms. *Limnol. Oceanogr.* **41**, 1344–1347 (1996).

19. C. S. Hopkinson, J. J. Vallino, A. Nolin, Decomposition of dissolved organic matter from the continental margin. *Deep. Res. Part II Top. Stud. Oceanogr.* **49**, 4461–4478 (2002).

20. Y. Shen, R. Benner, Mixing it up in the ocean carbon cycle and the removal of refractory dissolved organic carbon. *Sci. Rep.* **8**, 1–9 (2018).
