# Supplementary material for: Kelp carbon sink potential decreases with warming due to accelerating decomposition
Source: PLoS Biol. 2022 Aug 4;20(8):e3001702. doi: 10.1371/journal.pbio.3001702 (PMC9352061; doi:10.1371/journal.pbio.3001702)
Supplement: S2 Fig — δ13C in S. latissima (A) and L. hyperborea (B) and percent carbon content in S. latissima (C) and L. hyperborea (D) kelp detritus over the experiment. Data are frequency measures of ‰δ13C and %C from tissue samples taken at the onset of the experiment (T0), the first sampling time (T1), and the final sampling (T2). Y axes units are the proportion of observations. Measures are pooled across sites for each region and ordered by decreasing latitude. In some regions, insufficient tissue remained for T2 (Data B in S1 Data). (DOCX) [file pbio.3001702.s007.docx]

**
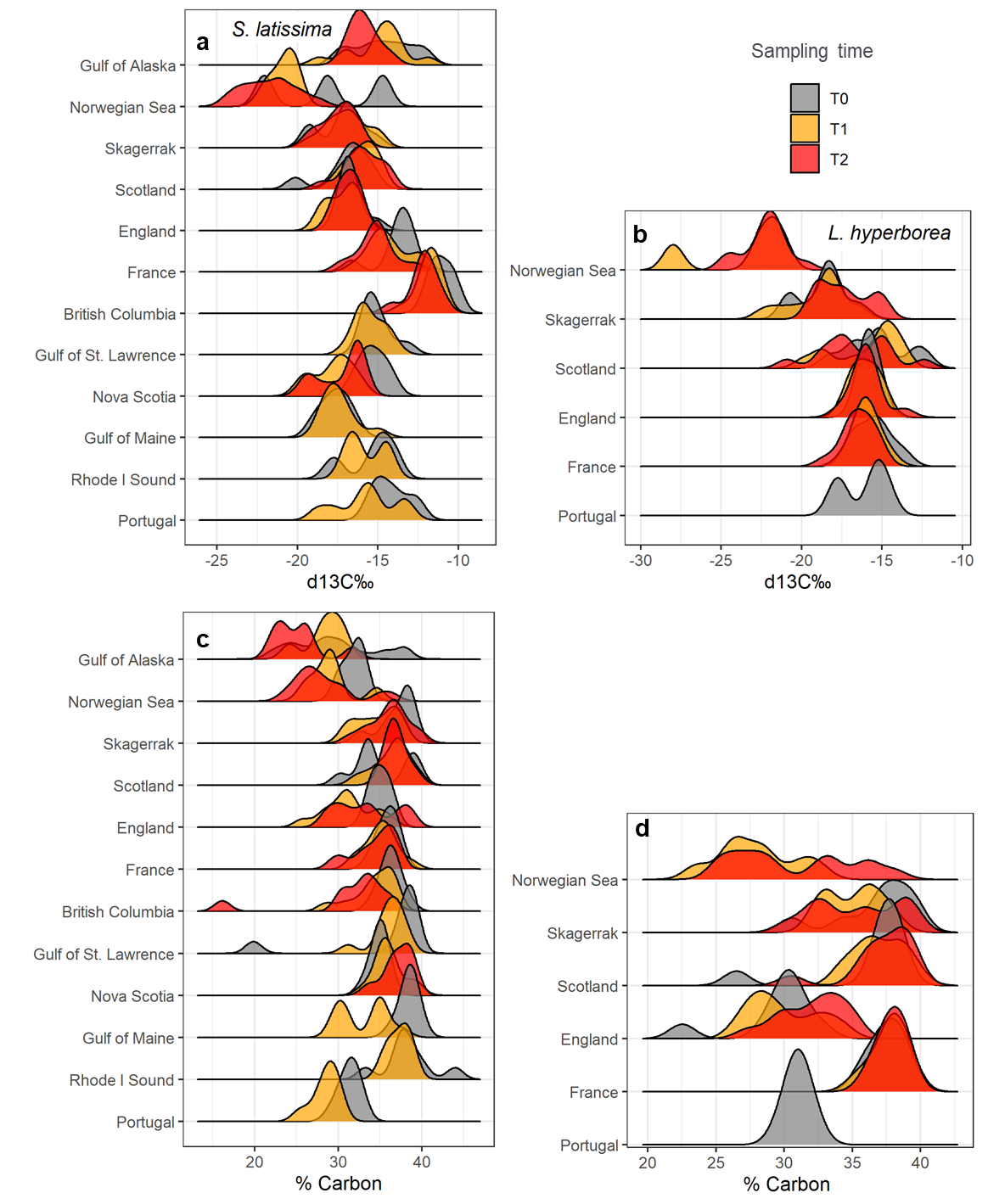
****S2 Fig. Change in carbon isotopic values and carbon content of kelp detritus**. δ^13^C in *S. latissima* (A) and *L. hyperborea* (B) kelp detritus and percent carbon content in *S. latissima* (C) and *L. hyperborea* (D) kelp detritus over the experiment. Data are frequency measures of ‰ δ^13^C and %C from tissue samples taken at the onset of the experiment (T0), the first sampling time (T1) and the final sampling (T2). Y axes units are the proportion of observations. Measures are pooled across sites for each region and ordered by decreasing latitude. In some regions insufficient tissue remained for T2.
