# Supplementary material for: Kelp carbon sink potential decreases with warming due to accelerating decomposition
Source: PLoS Biol. 2022 Aug 4;20(8):e3001702. doi: 10.1371/journal.pbio.3001702 (PMC9352061; doi:10.1371/journal.pbio.3001702)
Supplement: S3 Fig — Residence times (days to 50% decomposition) reported for different types of marine detritus, including kelps from our study regions (Sl = Saccharina latissima; Lh = Laminaria hyperborea) and measures reported in the literature for other seaweeds, seagrass, mangrove detritus (leaf), other POM and DOM (S5 Table). POM are from various sources, including zooplankton debris, feces, fauna casings, and marine snow. DOM are labile DOC or DOM released from zooplankton debris or marine snow during incubations. Refractory components of DOC are not included and residence times for these organic carbon pool can range from years to decades or more. DOC, dissolved organic carbon; DOM, dissolved organic material; POM, particulate organic material. (DOCX) [file pbio.3001702.s008.docx]

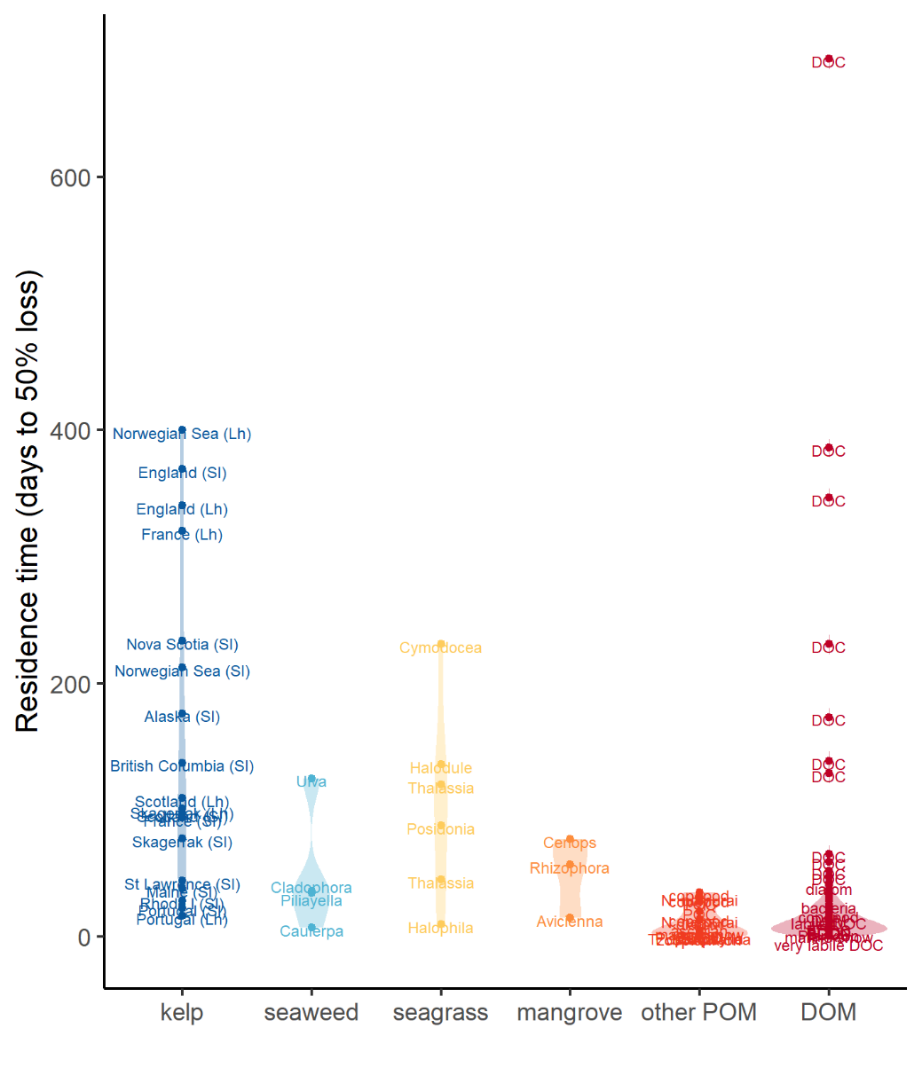


**S3 Fig. Residence time of marine detritus**. Residence times (days to 50% decomposition) reported for different types of marine detritus, including kelps from our study regions (Sl = *Saccharina latissima*; Lh = *Laminaria hyperborea*) and examples of measures reported in the literature for other seaweeds, seagrass, mangrove detritus (leaf), other particulate organic material (POM) and dissolved organic material (DOM) (S5 Table). POM are from various sources, including zooplankton debris, feces, fauna casings and marine snow. DOM are labile DOC or DOM released from zooplankton debris or marine snow during incubations. Refractory components of DOC are not included and residence times for these organic carbon pool can range from years to decades or more.
