# Supplementary material for: Kelp carbon sink potential decreases with warming due to accelerating decomposition
Source: PLoS Biol. 2022 Aug 4;20(8):e3001702. doi: 10.1371/journal.pbio.3001702 (PMC9352061; doi:10.1371/journal.pbio.3001702)
Supplement: S4 Fig — Average coastal residence times (days) simulated for each site location using global models of coastal residence time for water parcels exiting to the open ocean across the 200-m isobath (CRT) by Liu and colleagues (1) using the 0.125° resolution model, which was averaged over 1998–2007. Colors show study regions, ordered from left to right by decreasing latitude. Circles show average (±SE) site-level estimates of CRT. Crosses show average CRT for larger ecoregion (2) that the sites occurred (Data F in S1 Data). (DOCX) [file pbio.3001702.s009.docx]

**
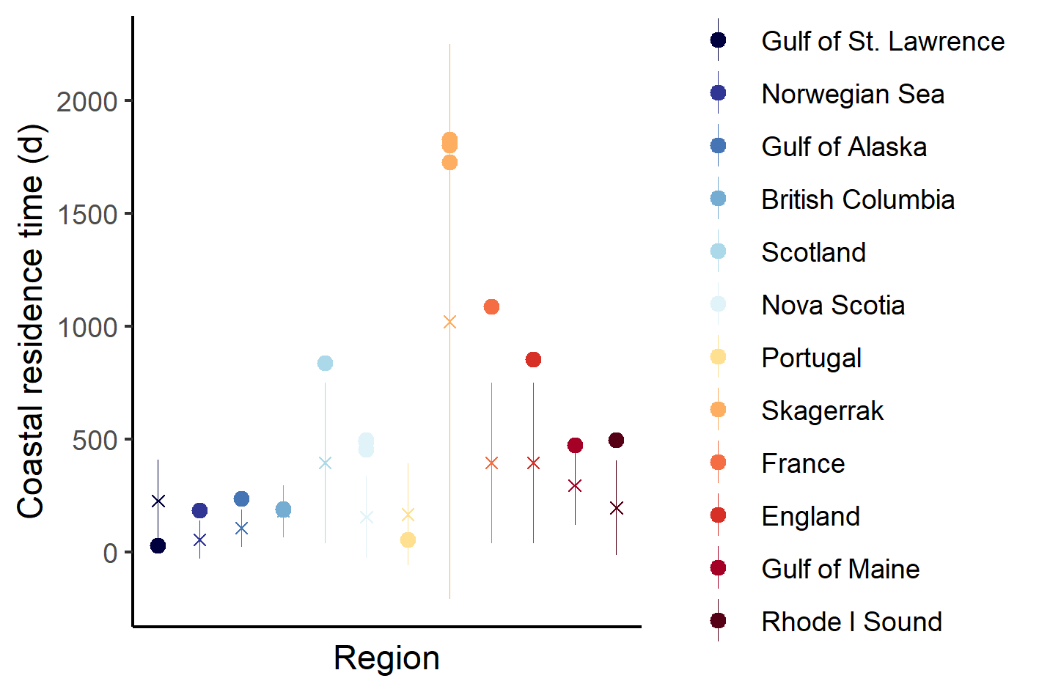
**

**S4 Fig.** **Coastal residence time**. Average coastal residence times (days) simulated for each site location using global models of coastal residence time for water parcels exiting to the open ocean across the 200-m isobath (CRT) by Liu et al. (1) using the 0.125° resolution model, which was averaged over 1998-2007. Colours show study regions, ordered from left to right by decreasing latitude. Circles show average (±SE) site-level estimates of CRT. Crosses show average CRT for larger ecoregion that the sites occurred (2).

**Reference**

1. X. Liu, *et al.*, Simulating Water Residence Time in the Coastal Ocean: A Global Perspective. *Geophys. Res. Lett.* **46**, 13910–13919 (2019).

2. M. D. Spalding, *et al.*, Marine ecoregions of the world: A bioregionalization of coastal and shelf areas. *Bioscience* **57**, 573–583 (2007).
