# Supplementary material for: Kelp carbon sink potential decreases with warming due to accelerating decomposition
Source: PLoS Biol. 2022 Aug 4;20(8):e3001702. doi: 10.1371/journal.pbio.3001702 (PMC9352061; doi:10.1371/journal.pbio.3001702)
Supplement: S1 File — (DOCX) [file pbio.3001702.s010.docx]

**SUPPLEMENTARY INFORMATION**

**Ocean temperature controls kelp decomposition and carbon sink potential**

Karen Filbee-Dexter*, Colette J. Feehan, Dan Smale, Kira A. Krumhansl, Skye Augustine, Florian de Bettignies, Michael T. Burrows, Jarrett E. K. Byrnes, Jillian Campbell, Dominique Davoult, Kenneth H. Dunton, João N. Franco, Ignacio Garrido, Sean P. Grace, Kasper Hancke, Ladd E. Johnson, Brenda Konar, Pippa J. Moore, Kjell Magnus Norderhaug, Alasdair O’Dell, Morten F. Pedersen, Anne K. Salomon, Isabel Sousa-Pinto, Scott Tiegs, Dara Yiu, Thomas Wernberg.

*Corresponding author: K Filbee-Dexter, kfilbeedexter@gmail.com

**Supplementary Information 1**

**Additional model and model fit assessments.**

Model fit assessments for GLMM, showing relationship between decomposition and temperature, species, temperature range and light. Residuals are visualized using DHARMa package (1). All predictor terms in these models were included in order to test specific hypotheses regarding their effects on kelp detritus decomposition. Because of the already large number of predictor variables we chose to not include interaction terms. This enabled us to present a full model with all hypothesized predictors, which had enough power to detect these relationships. This was appropriate because, based on the combined ecological knowledge of these systems from the author group, we did not identify a specific interaction important enough to warrant inclusion in our analysis as a specific hypothesis (2).


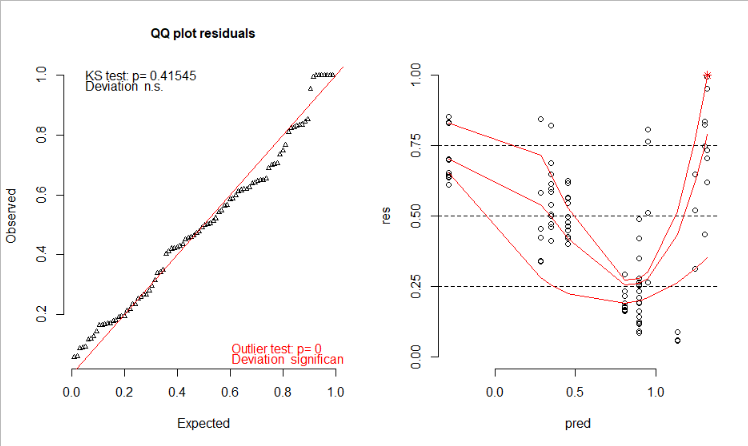


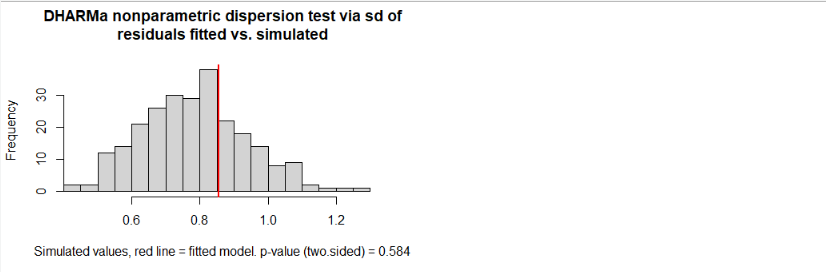


**Decomposition vs. latitude.** Linear mixed model fit by maximum likelihood.

Formula: WWperday ~ Latitude + (1 | Region/Site). *F* tests use Satterthwaite’s method.

| **Model 1**  **Fixed effects** | **DF** | **MS** | **F** | **p** |
| --- | --- | --- | --- | --- |
| All parameters |  |  |  |  |
| Latitude | 12 | 0.8009 | 7.70 | **0.0164** |
| **Random effects** | **N** | **Variance** | **SD** |  |
| (1 \| Site:Region) | 34 | 0.004 | 0.066 |  |
| (1 \| Region) | 12 | 0.327 | 0.572 |  |
| Residual |  | 0.104 | 0.0.32 |  |

**Nitrogen enrichments vs. environmental variables.** Linear mixed model fit by maximum

likelihood showing relationship between percent increase of nitrogen and environmental

variables. Formula: Nitrogen rate ~ Mean Temperature+Species+Exposure+(1|Region/Site). *F* tests use Satterthwaite’s method.

| **Model 1**  **Fixed effects** | **DF** | **MS** | **F** | **p** |
| --- | --- | --- | --- | --- |
| All parameters |  |  |  |  |
| Average temperature | 36.5 | 24.840 | 1.27 | 0.267 |
| Light | 36.3 | 31.164 | 1.59 | 0.215 |
| Species | 40.7 | 0.268 | 0.013 | 0.907 |
| Water movement | 45.0 | 0.0003 | 0.000 | 0.997 |
| **Random effects** | **N** | **Variance** | **SD** |  |
| (1 \| Site:Region) | 22 | 0.00 | 0.000 |  |
| (1 \| Region) | 8 | 24.53 | 4.953 |  |
| Residual |  | 18.64 | 4.318 |  |

**Carbon loss vs. environmental variables.** Linear mixed model fit by maximum likelihood showing relationship between percent loss of carbon and environmental variables. Formula: Carbon loss rate ~ Mean Temperature+Species+Exposure+(1|Region/Site). *F*  tests use Satterthwaite’s method.

| **Model 1**  **Fixed effects** | **DF** | **MS** | **F** | **p** |
| --- | --- | --- | --- | --- |
| All parameters |  |  |  |  |
| Average temperature | 15.7 | 55.8 | 0.039 | 0.845 |
| Light | 23.0 | 330.3 | 0.233 | 0.634 |
| Species | 42.9 | 151.2 | 0.107 | 0.746 |
| Water movement | 41.6 | 792.7 | 0.559 | 0.459 |
| **Random effects** | **N** | **Variance** | **SD** |  |
| (1 \| Site:Region) | 22 | 0.00 | 0.000 |  |
| (1 \| Region) | 8 | 24.53 | 4.953 |  |
| Residual |  | 18.64 | 4.318 |  |

**Reference**

1. F. Hartig, “DHARMa: Residual Diagnostics for Hierarchical (Multi-Level / Mixed) Regression Models” (2020).

2. L. S. Aiken, S. G. West, R. R. Reno, *Multiple regression: Testing and interpreting interactions* (SAGE Publications, 1991).
